# Light alcohol consumption has the potential to suppress hepatocellular injury and liver fibrosis in non-alcoholic fatty liver disease

**DOI:** 10.1371/journal.pone.0191026

**Published:** 2018-01-17

**Authors:** Kazutoshi Yamada, Eishiro Mizukoshi, Takuya Seike, Rika Horii, Masaaki Kitahara, Hajime Sunagozaka, Kuniaki Arai, Tatsuya Yamashita, Masao Honda, Shuichi Kaneko

**Affiliations:** Department of Gastroenterology, Graduate School of Medicine, Kanazawa University, Kanazawa, Ishikawa, Japan; University of Basque Country, SPAIN

## Abstract

**Background & aims:**

The modest consumption of alcohol has been reported to decrease the incidence of fatty liver or prevalence of steatohepatitis. In this study, we investigated the effect of light alcohol consumption on liver function and gene expression in patients with non-alcoholic fatty liver disease (NAFLD).

**Methods:**

The study group was formed of 178 patients diagnosed with non-alcoholic fatty liver disease, subclassified into two groups for analysis based on the daily alcohol consumption: non-alcohol group and light alcohol consumer group (≤20 g of ethanol/day). Clinical characteristics, liver histological features, gene expression, comprehensively analyzed using microarrays (BRB-Array tools), and molecular network were evaluated and compared between the two groups.

**Results:**

No significant differences in steatosis or inflammation score were noted among the groups. However, the ballooning and fibrosis scores were significantly lower in the light alcohol consumer group than in the non-alcohol group. Gene expression analysis revealed a marked inhibition of the pathways involved in the immune response in the light alcohol group compared to that in the non-alcohol group.

**Conclusions:**

Light alcohol consumption might suppress activity of non-alcoholic steatohepatitis by reducing gene expression levels involved in the immune response. This inhibition in gene expression was associated with a lowering of liver fibrosis and hepatocellular injury.

## Introduction

Non-alcoholic fatty liver disease (NAFLD) is one of the most common causes of chronic liver disease, with non-alcoholic steatohepatitis (NASH) being identified in approximately 20% of cases of NAFLD [1, 2]. NASH commonly progresses to liver cirrhosis, which itself is a high risk factor for the development of hepatocellular carcinoma and hepatic failure. Therefore, NAFLD is an important cause of liver-related death. NAFLD has also been closely associated with metabolic syndrome, which is characterized by obesity, diabetes mellitus, dyslipidemia and hypertension. Furthermore, NAFLD is also a risk factor for coronary heart disease [3, 4].

Alcohol-related liver disease is defined by a gradual change in liver function, including steatohepatitis and liver cirrhosis that can lead to liver-related death. Additionally, alcohol consumption is considered an exacerbating factor of metabolic syndrome and liver disease, with the effects of alcohol being mediated through the association between alcohol consumption and a high-fat diet or obesity [5,6]. Interestingly, however, an epidemiological review of health status data for a general population has demonstrated that the prevalence of metabolic syndrome is lower in individuals with light to moderate alcohol consumption than in those who do not consume alcohol [7]. Other studies have also reported a beneficial effect of modest alcohol intake in reducing the risk of a cardiovascular event [8]. Therefore, light alcohol consumption may provide a beneficial effect for the human health.

The association between NASH and light alcohol consumption is unclear, and there are some opinions even small amount of alcohol intake could exacerbate liver failure and, therefore, abstinence from alcohol is recommended for this clinical population. This recommendation is based on the following proposed causal pathway. Specifically, acetaldehyde, an intermediate by-product of alcohol, would increase intracellular concentrations of free fatty acids, through an inhibition of peroxisome proliferator-activated receptor α [9], resulting in an accumulation of triglyceride, via suppression microsomal triglyceride transfer protein expression [10], and increasing the production of reactive oxygen species and, therefore, oxidative stress on the mitochondria [11]. A postulated association between alcohol consumption and an increased risk for hepatocellular carcinoma in patients with NASH-related cirrhosis [12] has further strengthened the recommendation for abstinence from alcohol in patients with NASH. However, in reality, the effect of light alcohol consumption on the pathogenesis of liver disease is largely unknown.

A recent study reported a lower prevalence rate of NAFLD among individuals who consume a light amount of alcohol than among a population of non-alcohol consumers [13, 14]. Moreover, the prevalence of pathological hepatic features, indicative of possible active steatohepatitis, was lower in light alcohol consumers compared to that in non-alcohol consumers [15, 16]. Based on these results, we hypothesized that a modest consumption of alcohol could possibly improve the pathophysiology of NASH. Therefore, the aim of our study was to evaluate the relationship between the amount of daily alcohol intake and the clinical findings and pathophysiological changes in patients with NAFLD in an effort to clarify the effects of modest alcohol consumption in patients with fatty liver disease, including those with NAFLD. We included a comprehensive analysis of gene expression, using microarrays (BRB-Array tools), to evaluate effects at the level of the molecular network as well.

## Materials and methods

### Patients and laboratory testing

Prospective patients were selected from the group of patients diagnosed with fatty liver disease at the Graduate School of Medicine of the Kanazawa University Hospital, Japan, between 1998 and 2013. Among them, patients who were able to discuss their drinking history in detail were targeted. Fatty liver disease was detected by abdominal ultrasound examination and confirmed by ultrasound-guided percutaneous liver biopsy. Patients were screened on the following criteria to exclude other causes of liver disease: positive screening for hepatitis B surface antigen and hepatitis C virus antibody; pathological findings indicative of viral, autoimmune, genetic and drug-induced liver disease; and identification of hepatocellular carcinoma. Habitual alcohol drinkers or those with a drinking history, defined as an ethanol consumption level of >20 g per day, were excluded.

**Fig 1:**
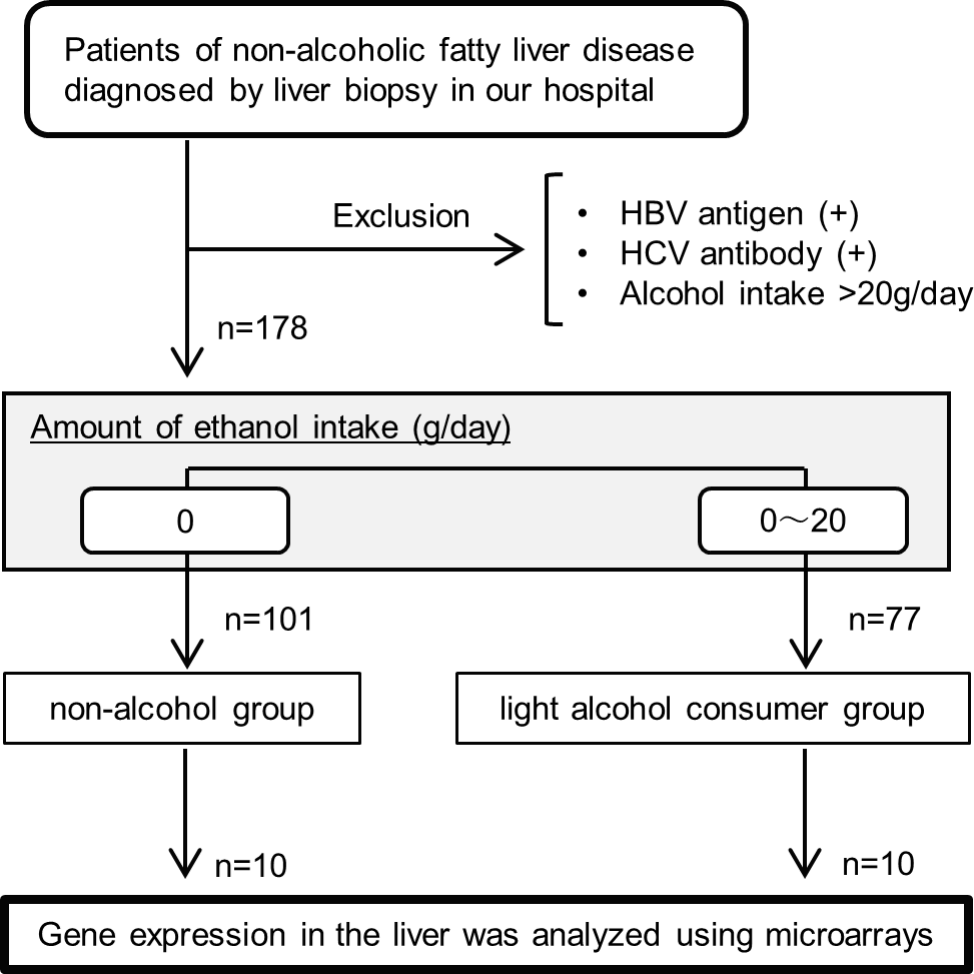
Inclusion and exclusion flow chart.

The study group was formed of 178 patients with NAFLD. Based on their self-reported habit of alcohol consumption, obtained at the time of admission for liver biopsy, patients were classified into two groups. One is non-alcohol group and the other is light alcohol consumer group, defined by an ethanol consumption ≤20 g per day. The flow diagram of patient identification and selection is shown in Fig 1.

All patients provided written informed consent to participate in the study in accordance with the Helsinki declaration and our study was approved by the regional ethics committee (Medical Ethics Committee of Kanazawa University, No. 1065).

Serum levels measured from samples obtained in a fasting state at the time of admission for liver biopsy were used in the analysis. A pathological evaluation was independently performed by pathologists who were blinded to the patients’ clinical information, including their volume of alcohol consumption, and scored them with respect to steatosis (0–3), lobular inflammation (0–3), hepatocellular ballooning (0–2), and fibrosis (0–4), according to the NASH Clinical Network Scoring System [17]. According to the NAFLD activity score (NAS), patients with 4 or fewer points were classified as simple steatosis, while those with 5 or more points were classified as NASH.

### Analysis using the Affymetrix Genechip

We completed a comprehensive, microarray analysis of gene expression in liver tissue for 10 patients, each, in the non- and light-alcohol groups. The quality of the isolated ribonucleic acid (RNA) was estimated after electrophoresis using an Agilent 2001 Bioanalyzer (Agilent, Santa Clara, CA). Aliquots (50 ng) of total RNA, isolated from the liver biopsy specimens, were subjected to amplification using the WT-Ovation Pico RNA Amplification System (NuGen, San Carlos, CA), according to the manufacturer's instructions. Approximately 10 μg of complementary deoxyribonucleic acid (cDNA) was amplified from the 50 ng of total RNA, with 5 μg of cDNA used for fragmentation and biotin labeling using the FL-Ovation cDNA Biotin Module V2 (NuGen), according to the manufacturer's instructions. Biotin-labeled cDNA was suspended in 220 μL of a hybridization cocktail (NuGen), with 200 μL of this suspension used for hybridization to the Affymetrix Human 133U Plus 2.0 GeneChip (Affymetrix, Santa Clara, CA) containing 54,675 probes. After stringent washing, the microarray chips were stained with streptavidin-phycoerythrin and probe hybridization was determined using a GeneChip Scanner 3000 (Affymetrix). Data files (CEL) were obtained using the GeneChip Operating Software 1.4 (Affymetrix).

### Hierarchical clustering and pathway analysis of the Genechip data

Analysis of the Genechip data was performed using the BRB-Array Tools (http://linus.nci.nih.gov/BRB-ArrayTools.htm). The data were log transformed, normalized, centered, and used in an average linkage hierarchical clustering analysis, with centered correlation.

Pathway analysis was performed using Biocarta, with functional ontology enrichment analysis performed to compare the distribution of gene ontology for differentially expressed genes, with a p < 0.05 used as a cut-off to determine difference in gene expression.

### Quantitative real time polymerase chain reaction (RT-PCR)

We performed quantitative RT-PCR using TaqMan Universal Master Mix (PE Applied Biosystems, Foster City, CA, USA) for patients who performed microarray analysis. Primer pairs and probes for toll like receptor (TLR) 4, signal transducer and activator of transcription 3 (STAT3), nuclear factor-kappa beta (NF-κB), CC chemokine ligand 5 (CCL5), Platelet endothelial cell adhesion molecule (Pecam1), cluster of differentiation (CD) 44, Proteasome subunit beta type-9 (PSMB9), intercellular adhesion molecule-1 (Icam1) and glyceraldehyde 3-phosphate dehydrogenase (GAPDH) were obtained from the TaqMan assay reagent library. Total RNA was isolated from liver tissue samples using an RNA extraction kit (Micro RNA Extraction Kit, Stratagene, La Jolla, CA, USA). We reverse-transcribed 1 g of isolated RNA to cDNA using SuperScript® II RT (Invitrogen, Carlsbad, CA, USA) according to the manufacturer’s instructions, and the resultant cDNA was amplified with appropriate TaqMan assay reagents as previously described [18].

### Statistical analysis

Data were expressed as the mean±standard deviation (SD). Differences in the clinical features and histopathological findings between the non- and light-alcohol consumer groups was evaluated using a Mann Whitney’s U test for continuous variables, Fisher's exact probability test for categorical variables and Logistic regression analysis for multivariate models. A p < 0.05 was considered significant.

## Results

### Patient profiles

Baseline characteristics of patients forming the study group are reported in Table 1. The mean age for the non-alcohol group was 53.1 years (17–88 years), with 39.6% of the group being males. The mean age for the light alcohol consumer group was 46.1 years (21–78 years), with 67.5% of the group males. In the light alcohol consumer group, a significant increase in prothrombin time activity and decrease in hyaluronic acid and type III procollagen-N-peptide (P-III-P) levels were identified compared to the non-alcohol groups. On the other hand, body mass index (BMI), aspartate transaminase (AST), alanine transaminase (ALT), Hemoglobin A1c, high density lipoprotein (HDL) cholesterol and low density lipoprotein (LDL) cholesterol, which are markers of obesity, diabetes, dyslipidemia, and liver enzymes, were comparable between the light- and non-alcohol groups. The prevalence of NAFLD-related complications, namely obesity, type II diabetes mellitus (DM), dyslipidemia, and hypertension, were comparable between the light- and non-alcohol groups.

**Table 1:** Clinical features of the 178 patients with fatty liver disease.

variable	NA (n = 101)	LA (n = 77)	*p*-value
Sex M/F	40/61	53/24	0.0003
Age (years)	53.1±17.2	46.1±14.4	0.0046
Smoking (yes/no)	27/74	43/34	0.0002
BMI (kg/m^2^)	28.8±5.26	29.4±7.07	0.8636
AST (IU/l)	46.1±42.6	40.1±22.9	0.6993
ALT (IU/l)	64.1±79.8	68.5±49.8	0.0610
ALP (IU/l)	276.4±101.4	248.6±76.8	0.0604
γ-GTP (IU/l)	82.7±72.0	69.0±53.2	0.4656
Platelet (×10^4^/mm^2^)	21.4±7.0	24.5±25.0	0.7246
Albumin (g/dl)	4.30±0.46	4.47±0.39	0.0050
Prothrombin time activity (%)	94.1±14.1	100.8±15.8	0.0024
Hemoglobin A1c (%)	7.00±1.77	6.75±1.91	0.1995
HOMA-IR	4.37±3.68	4.91±5.50	0.2837
Fasting blood glucose (mg/dl)	130.4±39.2	121.3±46.8	0.0405
Total cholesterol (mg/dl)	189.3±39.4	193.2±39.8	0.6639
Triglycerides (mg/dl)	143.3±84.3	138.7±67.2	0.7871
HDL cholesterol (mg/dl)	45.4±11.5	45.7±11.6	0.9574
LDL cholesterol (mg/dl)	115.8±34.8	119.5±37.1	0.5744
P-β-P (U/ml)	0.69±0.25	0.58±0.18	0.0070
Hyaluronic acid (ng/ml)	81.5±138.8	38.1±43.3	0.0726
Type IV collagen (ng/ml)	5.20±2.17	4.25±1.74	0.0006
Ferritin (ng/ml)	197.7±327.5	260.2±248.2	0.0374
Obesity*	72 (71.3%)	54 (70.1%)	0.8696
Diabetes**	74 (73.3%)	46 (59.7%)	0.0755
Dyslipidemia***	64 (63.4%)	49 (63.6%)	>0.9999
Hypertension	42 (41.6%)	24 (31.2%)	0.1627

Notes: The data are expressed as mean±SD; NA, non-alcohol group; LA, light alcohol consumer group; BMI, body mass index; AST, aspartate transaminase; ALT, alanine transaminase; ALP, alkaline phosphatase; γ-GTP, γ-glutamyltransferase; HOMA-IR, homeostasis model assessment-Insulin Resistance; HDL, high density lipoprotein; LDL, low density lipoprotein; P-III-P, type III procollagen-N-peptide.

*: BMI ≥ 25

**: HbA1c ≥ 6.5 or medication therapy was started

***: LDL cholesterol ≥ 140 mg/dl, triglycerides ≥ 150 mg/dl, or medication therapy was started.

### Histopathological findings

Histopathological findings are summarized in Table 2. The proportion of patients with NASH was lower in the light alcohol group than in the non-alcohol group. Although steatosis and lobular inflammation scores were comparable among the groups, the ballooning and fibrosis scores were significantly lower in the light alcohol consumer group than in the non-alcohol group. (representative histological images are shown in S1 Fig). Therefore, the light alcohol consumer group exhibited a lower steatohepatitis grade than the non-alcohol group.

**Table 2:** Histopathologic findings of liver in the study population.

		NA (n = 101)	LA (n = 77)	Adjusted OR (95% CI)	*p*-value
NAFLD activity score	4≤	54	46	0.752 (0.397–1.427)	0.384
	5≥	47	31		
Steatosis score	1	43	35	0.847 (0.560–1.282)	0.433
	2	34	21		
	3	24	21		
Lobular inflammation score	0	5	6	0.964 (0.620–1.500)	0.871
	1	44	29		
	2	44	38		
	3	8	4		
Hepatocellularballooning score	0	37	39	0.575 (0.364–0.907)	0.017
	1	37	33		
	2	27	5		
Fibrosis score	0	5	12	0.707 (0.512–0.977)	0.035
	1	55	45		
	2	15	7		
	3	14	11		
	4	12	2		

Multivariate models adjusted for gender and age.

Notes: NA, non-alcohol group; LA, light alcohol consumer group

### Microarray analysis

**Fig 2:**
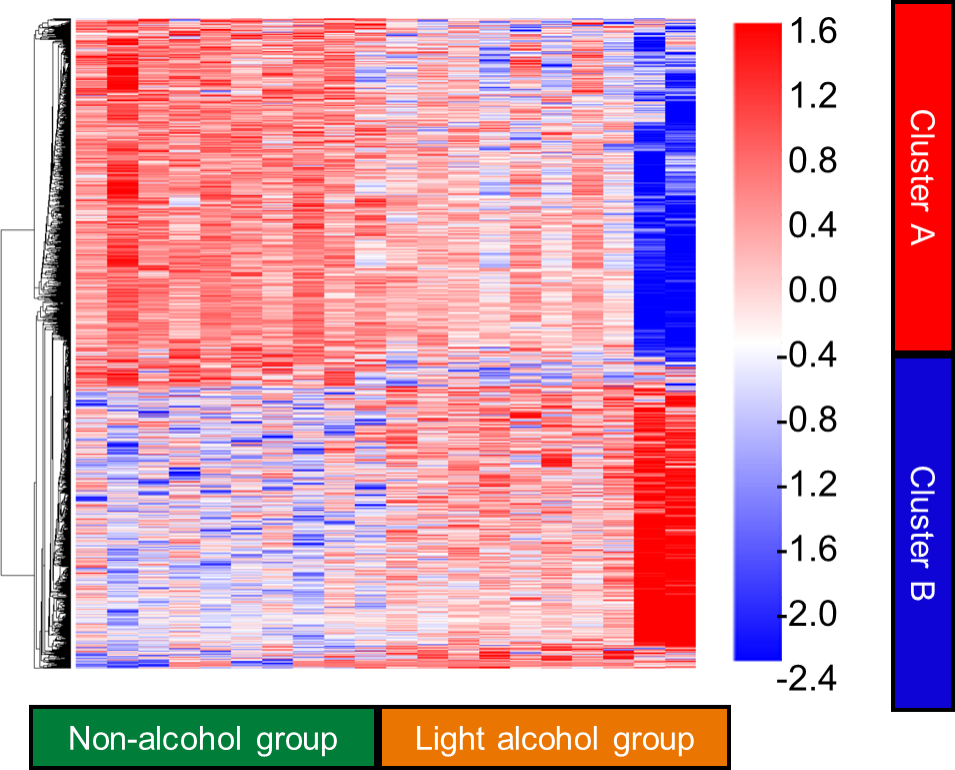
Hierarchical clustering of expression in non-alcohol group and light alcohol consumer group. In non-alcohol group, 848 genes (Cluster A) and 462 genes (Cluster B) were up-regulated and down-regulated, respectively. Each cell in the matrix represents the expression of a gene in an individual sample. Red and blue cells depict high and low expression levels, respectively, as indicated by the scale bar (fold change in expression). Non-alcohol group and light alcohol consumer group are depicted as green and yellow boxes, respectively.

Subjects with the microarray analysis of gene were randomly selected from patients who could be obtained enough liver samples to extraction of the RNA. The clinical characteristics of patients for whom a microarray analysis was performed are summarized in Table 3. Age, ALT levels and histopathological findings were comparable between the light- and non-alcohol groups. In this analysis, we identified a total of 1310 genes that were differentially expressed between non-alcohol group and light alcohol group. Of these genes, 848 were down-regulated, (the representative genes were CD14, CD44 and ICAM1, shown in S1 Table) and 462 were up-regulated in the light alcohol consumer group. By using a hierarchical cluster analysis, these two group was divided clearly (Fig 2). The 8 first pathways, based on the BioCarta pathway database, are reported in Table 4 for the light- and non-alcohol groups. The following between-group differences were identified. The pathways involved in the immune response were markedly inhibited in the light alcohol group compared to those in the non-alcohol group, with activity of the monocyte, and its surface molecules, pathway being particularly reduced in the light alcohol consumer group.

**Table 3:** Characteristics of patients included in the cDNA microarray analysis.

Number	Group	Age	Sex	DM	DL	HT	ALT	BMI	Fibrosis	Steatosis	Inflammation	Ballooning
1	NA	52	F	+	+	-	36	31.3	1	1	2	0
2	NA	48	M	+	+	+	89	28.8	1	3	1	0
3	NA	24	M	+	+	-	191	27.5	1	3	2	0
4	NA	36	F	+	+	-	89	23.5	1	3	1	0
5	NA	53	M	+	-	+	19	22.0	1	1	1	0
6	NA	34	F	+	+	-	87	23.6	1	3	1	1
7	NA	68	F	+	+	+	33	23.6	1	1	1	1
8	NA	46	M	+	+	+	87	40.5	1	2	1	2
9	NA	65	M	-	+	+	102	29.5	1	2	3	1
10	NA	29	M	+	+	+	123	38.7	1	3	2	1
Average		45.5					85.6	28.9	1	2.2	1.5	0.6
11	LA	34	M	+	+	+	85	42.6	1	3	2	0
12	LA	54	M	-	+	-	45	23.6	1	1	1	0
13	LA	40	M	-	+	-	59	32.4	0	1	2	0
14	LA	61	M	+	-	-	68	22.1	1	1	1	0
15	LA	59	M	-	+	+	45	25.3	0	1	1	0
16	LA	65	M	+	+	-	57	23.7	1	3	1	1
17	LA	78	M	+	+	+	19	22.7	1	2	1	2
18	LA	40	M	+	-	+	38	41.4	2	2	1	0
19	LA	37	M	-	+	+	62	33.1	2	3	3	0
20	LA	22	M	-	-	-	127	27.0	1	3	2	1
Average		49					60.5	29.4	1	2	1.5	0.4

Notes: ALT, alanine aminotransferase; BMI, body-mass index; DL, dyslipidemia; DM, diabetes mellitus; F, female; HT, hypertension; LA, light alcohol consumer group; NA, non-alcohol group; M, male; F, female.

**Table 4:** Between-group comparison of the up- and down-regulated pathways, by gene set, for the non-alcohol group and light alcohol consumer group (BRB-array tool).

Pathway	No. of genes	LS p value	KS p value
**Down-regulated in light alcohol consumer group**			
The Role of Eosinophils in the Chemokine Network of Allergy	10	0.00001	0.01879
Monocyte and its Surface Molecules	30	0.00003	0.0053
Antigen Processing and Presentation	23	0.0002	0.02815
B Lymphocyte Cell Surface Molecules	22	0.00022	0.01653
Adhesion Molecules on Lymphocyte	29	0.00023	0.00885
Neutrophil and Its Surface Molecules	19	0.00029	0.02593
**Up-regulated in light alcohol consumer group**			
uCalpain and friends in Cell spread	35	0.00367	0.00018
Neuropeptides VIP and PACAP inhibit the apoptosis of activated T cells	54	0.00954	0.00002

### RT-PCR

**Fig 3:**
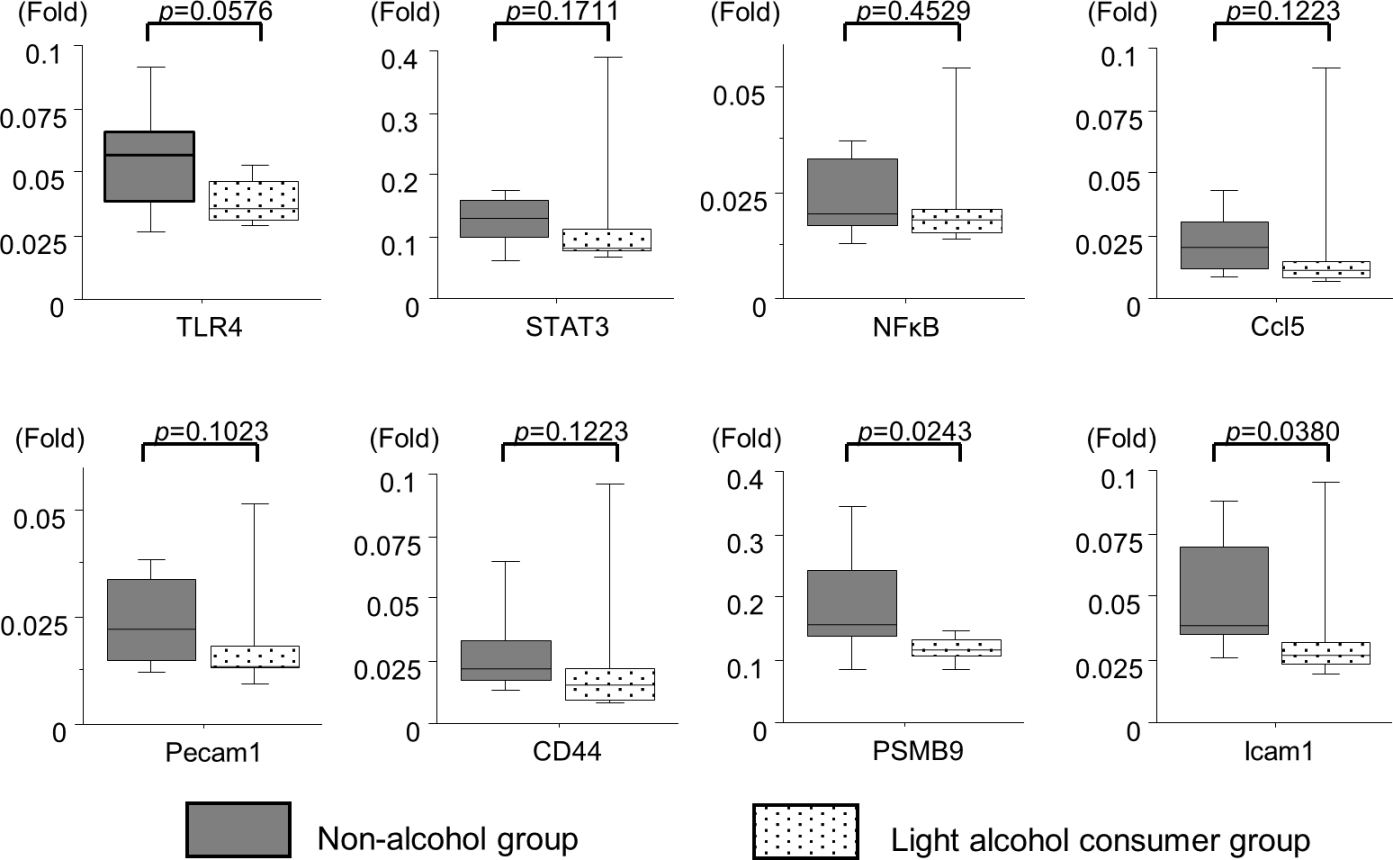
Hepatic gene expression levels involved in immune response pathway or TLR4 signaling. In 20 patients (non-alcohol group: 10, light alcohol consumer group: 10), the expression levels of genes were measured using RT-PCR, and evaluated using the Mann-Whitney U test. The expression levels of PSMB9 and Icam1 were significantly higher in the non-alcohol group than in the light alcohol consumer group. There was also a trend of decreased expression levels of TLR4 and other genes among patients in the light alcohol consumer group. Notes: TLR4, toll like receptor (TLR) 4; STAT3, signal transducer and activator of transcription 3; NFκB, nuclear factor-kappa beta; Ccl5, CC chemokine ligand 5; Pecam1, Platelet endothelial cell adhesion molecule; CD44, cluster of differentiation 44; PSMB9,Proteasome subunit beta type-9 (PSMB9); Icam1, intercellular adhesion molecule-1.

To support the array findings, the expression levels of some genes involved in the immune response pathway or TLR4 signaling were investigated. Samples of 20 (non-alcohol group: 10, light alcohol consumer group: 10) patients (same as the number of patients included in the microarray analysis) were subjected to RT-PCR, and the expression levels of eight genes—TLR4, STAT3, NFκB, Ccl5, Pecam1, CD44, PSMB9, and Icam1—were measured. The results are shown in Fig 3. The expression levels of PSMB9 and Icam1 were significantly higher in the non-alcohol group than in the light alcohol consumer group. With respect to the other genes, there was a trend of increased expression levels among patients in the non-alcohol group.

## Discussion

Our study provides evidence of lower hepatocellular damage and liver fibrosis in patients with NAFLD who habitually consume a light amount of alcohol, compared to that in a group of patients with NAFLD who did not consume alcohol. There were no differences in the rate of obesity, DM, dyslipidemia and hypertension or clinical markers of liver pathophysiology, of clinical data associated with NASH, including BMI and levels of aminotransferase, triglycerides, cholesterol and hemoglobin A1c, between the light- and non-alcohol groups. Therefore, a small, daily intake of alcohol appears to confer a potentially beneficial effect on the state of steatohepatitis.

To date, a definite conclusion on the effects of light or moderate daily alcohol consumption in patients with NAFLD is not available, with both favorable and negative opinions having been expressed. However, most cross-sectional studies conducted on this topic have reported some degree of a protective effect of regular alcohol consumption on NAFLD. Specifically, modest alcohol consumption was shown to lower levels of aminotransferase [13] and the prevalence of fatty liver disease [14,19], as well to reduce levels of fibrosis and hepatocellular ballooning. In addition, a meta-analysis of 43,175 subjects has provided evidence of the positive effect of modest alcohol consumption in lowering the risk for NAFLD [20]. Although there is increasing evidence of a beneficial effect of modest alcohol intake on health outcomes, including liver function, support from basic medical research and prospective studies are needed to inform practice.

Currently, there are more reports providing a negative opinion on alcohol consumption in patients with NASH [21]. A basic science study using a mice model demonstrated that the combination of alcohol intake and a high-fat diet worsened steatohepatitis status, compared to either alcohol intake or a high-fat diet independently [22]. This study, however, did not evaluate the effects of small to modest amounts of alcohol intake. The retrospective study by Ascha et al. [12] provided evidence on an increased risk for hepatocellular carcinoma in patients with underlying NASH who habitually consumed alcohol. However, it is important to note that Ascha et al.’s study was limited to patients with advanced liver fibrosis and, therefore, the effects of light alcohol consumption in patients with NAFLD or early stages of NASH remains to be clearly defined. This underlines the importance of our study in providing a new way forward to investigate the effect of light alcohol intake in patients with NAFLD.

To our knowledge, we are the first to have evaluated the effects of light alcohol consumption on gene expression in the liver, as well as on clinical and histological features of NAFLD. Comparing gene expression in 10 patients in the light alcohol group and 10 patients in the non-alcohol group, we identified clear between-group differences in gene expression using a hierarchical cluster analysis. (Fig 2) Specifically, we identified an inhibition of the immune response pathway with light alcohol consumption, which might improve the pathological condition of NAFLD. However, the current study has several limitations. Age and gender were not equivalent between the two groups, and the subset of patients used for the gene array analysis was not the best representative sample of the original grouping. These are aspects that need to be discussed.

A relationship between immune response and NAFLD/NASH has been previously proposed, although the immunological factors involved have not been clearly defined. It has been shown that hepatic enrichment with natural killer T cells is a relevant factor to the progression of fibrosis and increasing the NAS in patients with NASH [23, 24]. As well, with over-nutrition, the M1 pro-inflammatory cytokines have been shown to promote the development of steatosis via changes in metabolic pathways of biliary lipids secreted by the hepatocytes [25]. Liver injury and fibrosis has also been linked to an increase in tumor necrosis factor α (TNF-α), with the TNF-α possibly being induced by liver macrophages [26]. Therefore, the involvement of immunocompetent cells is considered to play some role in the progression of inflammation and fibrosis of liver tissue in the disease process of NASH.

Recently, there has been increasing evidence of an association between adipose tissue in obesity and chronic inflammation, characterized by abnormal cytokine production [27]. The recruitment of macrophages in adipose tissue, and the subsequent chronic inflammation induced by endogenous TLR ligands, is considered an important mechanism of metabolic syndrome [28]. In a similar way, increased TLR4 signaling and activation of macrophage is considered to be one of the causes of the development of steatohepatitis. Interestingly, increased TLR4 signaling has also been associated with alcoholic steatohepatitis. In the case of alcohol consumption, the macrophage-derived inflammatory cytokines would be induced by an increase in the level of endotoxins from intestinal bacteria due to the alcohol-related increase in intestinal permeability. The increase in macrophage-derived inflammatory cytokines from this pathway is considered to be an important factor of hepatic inflammation in patients with alcoholic hepatitis [29, 30]. This macrophage activation, via TLR4 signaling, and subsequent increase of inflammatory cytokines is a common mechanism of NASH and alcoholic steatohepatitis. This evidence has supported the view that alcohol intake would increase the pathology of NASH by increasing the level of inflammatory cytokines via an increase in TRL4 signaling.

In contrast to these findings, our microarray analysis data showed the CD14 expression was significantly lower among patients in the light alcohol consumer group (*p* < 0.05). There was also a trend to a decreased expression of TLR4 or STAT3 among patients in the light alcohol consumer group. (data is not shown) Additionally, the hepatic expression level of TLR4 confirmed by RT-PCR showed a decreasing trend in the light alcohol consumer group as compared with that in the non-alcohol group. It is important to consider that a light alcohol intake, defined by ≤20 g of alcohol per day, may not be sufficient to increase levels of gut-derived lipopolysaccharides (LPS), which are found in individuals with a habitual heavy intake of alcohol (>60 g per day). That LPS levels correlate with the amount of alcohol intake has been previously confirmed, our study provides novel evidence that light alcohol intake, defined by ≤20 g of alcohol per day, may not be sufficient to increase levels of LPS. It also plausible that a light daily intake of alcohol may be effective in inhibiting immune responses and, thereby, controlling the disease activity in NASH. This inflammatory-suppression role of alcohol has been previously described. In vitro studies have shown that pre-exposure to a moderate level of alcohol enhances the tolerance of monocytes and macrophages to endotoxins, as well as reducing NF-κB promoter activity, induced by LPS, and, therefore, the downstream production of TNF-α, interleukin (IL) 6 and IL-1β [31]. Moreover, alcohol consumption changes the phenotype of macrophages from M1 to M2, which further helps to resolve an inflammatory state [32]. In the present study, serum level of TNF-α decreased in individuals in the light alcohol consumer group, however serum level of endotoxin was no significantly difference between the two groups. (S2 Fig). These effects of light alcohol intake on the inflammatory response pathway would oppose the pathogenesis of NASH and improve outcomes for patients.

We also identified a strong suppression of monocytes and their surface molecules in the light alcohol consumer group compared to that in the non-alcohol group, indicative that a small amount of alcohol intake may be effective in suppressing macrophage-derived pro-inflammatory cytokines and, thereby, delaying the progression of steatohepatitis. Therefore, the increase in inflammatory cytokines that occurs due to heavy alcohol intake does not occur with a light alcohol intake, favoring immunosuppression and a reduction in inflammatory cytokines. Therefore, depending on the amount of intake, alcohol can either be a main cause of liver failure or offer a protective effect against chronic liver inflammation. It is interesting to note that a long-term moderate consumption of alcohol has been shown to lower the risk for rheumatoid arthritis risk by reducing the level of inflammatory cytokines [33].

In conclusion, light alcohol consumption in patients with NAFLD was associated improved pathological features of NASH, hepatocellular ballooning and fibrosis of the liver. Beneficial effects of light alcohol consumption were further identified at the level of gene expression in liver tissue, with evidence of inhibition of the inflammatory pathway. Therefore, modest alcohol consumption could play a role in inhibiting the progression to NASH in patients with NAFLD.

## Supporting information

S1 Fig Histological images for the non-alcohol group and light alcohol consumer group. (A) Liver sample of non-alcohol group: age 57, male, Fibrosis 3, Steatosis 2, Lobular inflammation 2, Ballooning 1. (B) Liver sample of light alcohol consumer group: age 58, male, Fibrosis 1, Steatosis 2, Lobular inflammation 2, Ballooning 0.
(TIF)

S2 Fig Serum levels of TNF-α and endotoxin. We investigated the serum levels of TNF-α and endotoxin in patients (52 patients in the non-alcohol group and 41 patients in the light alcohol consumer group). Detection sensitivity of endotoxin was 1 pg / ml or more, and below detection sensitivity was analyzed as 1 pg / ml.
(TIF)

S1 Table Genes differentially expressed in non-alcohol consumption group and light alcohol consumption group. (PDF)
