# Supplementary figures and images for: Light alcohol consumption has the potential to suppress hepatocellular injury and liver fibrosis in non-alcoholic fatty liver disease
Source: PLoS One. 2018 Jan 17;13(1):e0191026. doi: 10.1371/journal.pone.0191026 (PMC5771612; doi:10.1371/journal.pone.0191026)

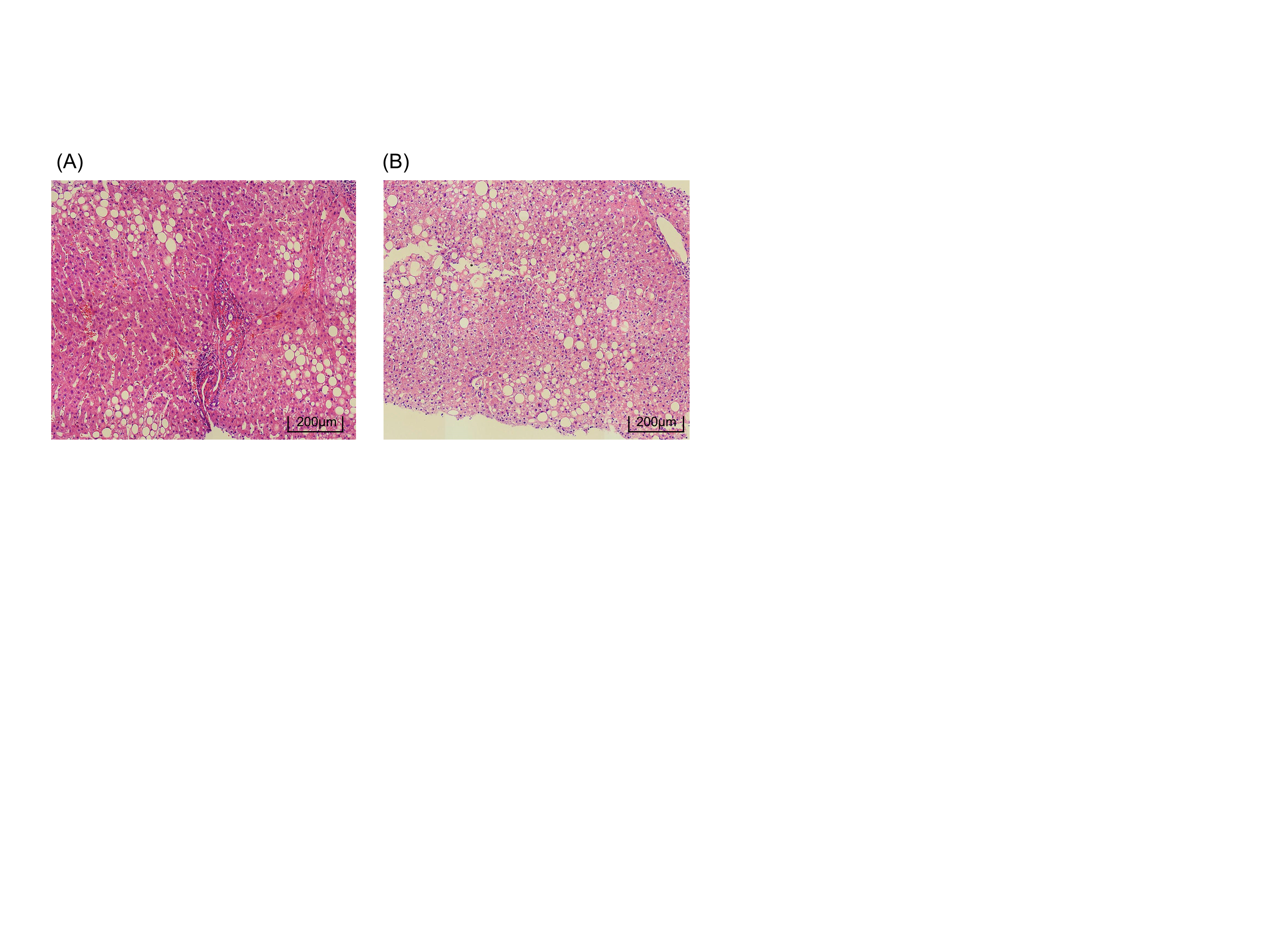

Supplement: S1 Fig — (A) Liver sample of non-alcohol group: age 57, male, Fibrosis 3, Steatosis 2, Lobular inflammation 2, Ballooning 1. (B) Liver sample of light alcohol consumer group: age 58, male, Fibrosis 1, Steatosis 2, Lobular inflammation 2, Ballooning 0. (TIF) [file pone.0191026.s001.TIF]

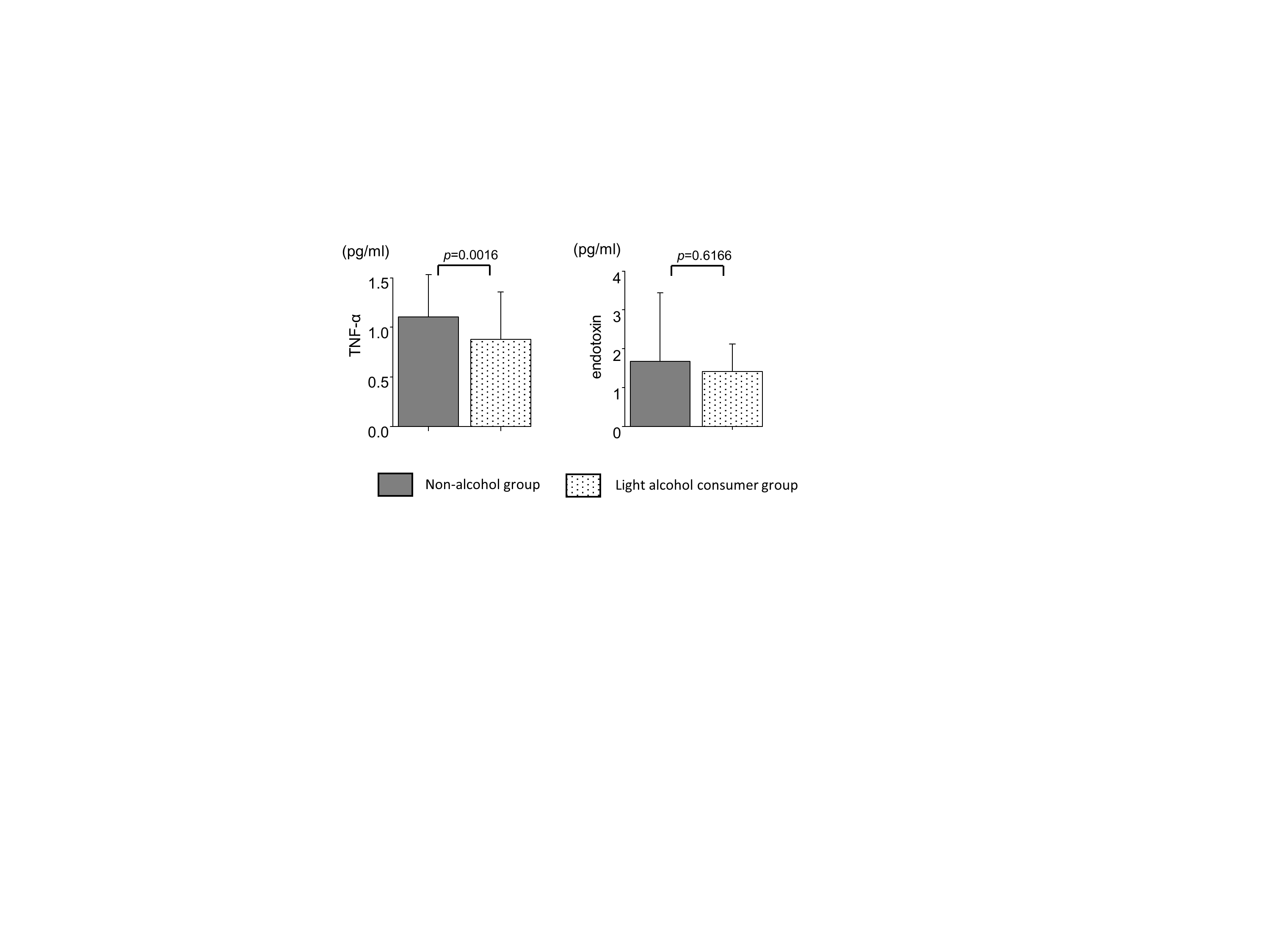

Supplement: S2 Fig — We investigated the serum levels of TNF-α and endotoxin in patients (52 patients in the non-alcohol group and 41 patients in the light alcohol consumer group). Detection sensitivity of endotoxin was 1 pg / ml or more, and below detection sensitivity was analyzed as 1 pg / ml. (TIF) [file pone.0191026.s002.TIF]
