# Supplementary material for: Light alcohol consumption has the potential to suppress hepatocellular injury and liver fibrosis in non-alcoholic fatty liver disease
Source: PLoS One. 2018 Jan 17;13(1):e0191026. doi: 10.1371/journal.pone.0191026 (PMC5771612; doi:10.1371/journal.pone.0191026)
Supplement: S1 Table — (PDF) [file pone.0191026.s003.pdf]

Supplementary Table 1. Genes differentially expressed in non-alcohol consumption group and light alcohol consumption group.

Parametric *P*-value	FDR	Fold-change (ND/LD*)	Gene Symbol	UG Cluster
9.53E-05	0.473	0.68	BAGE	Hs.545789
0.0003243	0.473	1.5	FAM96B	Hs.9825
0.0003837	0.473	0.65	DSCC1	Hs.315167
0.0005377	0.473	1.61	PARP8	Hs.369581
0.0006533	0.473	1.3	ARHGAP5	Hs.592313
0.0006787	0.473	1.6	KDM6A	Hs.522616
0.0006833	0.473	2.08	GPR82	Hs.567457
0.0006899	0.473	1.29	EFEMP2	Hs.731454
0.0010572	0.473	0.65	PDC	Hs.654381
0.0010924	0.473	1.9	GZMA	Hs.90708
0.0011552	0.473	1.78	LOC100506392	Hs.418285
0.0011565	0.473	0.64	ETV3	Hs.105636
0.0012719	0.473	1.95	TMEM45B	Hs.504301
0.001323	0.473	1.24	RNF10	Hs.442798
0.0015253	0.473	0.66	FBXL18	Hs.623974
0.0015912	0.473	0.65	INTS5	Hs.458390
0.0016191	0.473	0.71	STAMBP	Hs.469018
0.0016702	0.473	2.09	TOX3	Hs.460789
0.0016828	0.473	0.72	TECR	Hs.515642
0.0019103	0.473	1.4	CCNDBP1	Hs.36794
0.0020093	0.473	2.04	MBOAT1	Hs.377830
0.0020646	0.473	2.19	FCGR2A	Hs.352642
0.0021142	0.473	1.59	ZCCHC7	Hs.654700
0.0022842	0.473	1.4	DEF8	Hs.62771
0.0023629	0.473	1.33	RECQL	Hs.235069
0.002484	0.473	2.41	LOX	Hs.102267
0.0025964	0.473	1.52	ARHGEF2	Hs.743352
0.0026707	0.473	0.75	DHX30	Hs.517948
0.0026965	0.473	1.57	CCDC3	Hs.498720
0.0027793	0.473	0.64	LOC100506379	Hs.732759
0.0029407	0.473	1.34	ZNF689	Hs.454685

0.0029719	0.473	2.02	CCL5	Hs.514821
0.0033183	0.473	0.75	TFDP1	Hs.79353
0.0033225	0.473	0.44	KIAA0101	Hs.81892
0.0033751	0.473	0.79	NOL10	Hs.222494
0.0033814	0.473	0.71	BANP	Hs.461705
0.0034195	0.473	0.65	ULK4	Hs.656192
0.0034527	0.473	1.33	ELMSAN1	Hs.743376
0.0034631	0.473	1.43	TWSG1	Hs.514685
0.0034952	0.473	1.73	CREBRF	Hs.484195
0.0036875	0.473	0.57	FMO2	Hs.144912
0.0038581	0.473	1.88	TATDN3	Hs.530538
0.0038875	0.473	1.85	IPO4	Hs.411865
0.0038996	0.473	1.58	GBP5	Hs.513726
0.0039	0.473	1.22	TTBK2	Hs.646511
0.0039365	0.473	0.53	NPR1	Hs.490330
0.003941	0.473	2.06	GAS1	Hs.65029
0.0040977	0.473	1.88	GRIA3	Hs.377070
0.0041764	0.473	0.64	SNHG10	Hs.448753
0.0042483	0.473	0.68	MAOB	Hs.654473
0.0042649	0.473	0.67	GTPBP5	Hs.340636
0.0042886	0.473	0.66	USP30-AS1	Hs.660996
0.0044279	0.473	1.41	SLC16A1	Hs.75231
0.0045768	0.473	1.78	ZNF426	Hs.594011
0.0046244	0.473	0.73	DLGAP4	Hs.249600
0.0047691	0.473	1.23	OPA3	Hs.466945
0.0047849	0.473	1.37	TAP1	Hs.352018
0.0047952	0.473	1.35	SLC44A1	Hs.573495
0.0048482	0.473	1.88	CXCR6	Hs.34526
0.005082	0.473	1.54	SRGAP2C	Hs.497575
0.0050876	0.473	2.06	C16orf54	Hs.331095
0.0051903	0.473	0.59	TMEM63A	Hs.119387
0.0052019	0.473	0.74	SCMH1	Hs.571874
0.0052359	0.473	1.86	STK17B	Hs.88297
0.0053391	0.473	2.32	ANXA2	Hs.511605
0.0053809	0.473	1.65	RAB8B	Hs.389733
0.0055096	0.473	1.49	HES1	Hs.250666

0.0055518	0.473	1.47	SRGAP2	Hs.497575
0.005594	0.473	1.37	KLF11	Hs.12229
0.0056113	0.473	0.58	ZNF345	Hs.362324
0.0056139	0.473	2.17	C7	Hs.78065
0.0057301	0.473	1.86	ABCC4	Hs.508423
0.0058242	0.473	1.76	OFD1	Hs.6483
0.0058724	0.473	0.72	DCTN4	Hs.675564
0.0058901	0.473	1.86	TRIM47	Hs.293660
0.0059726	0.473	0.82	PAXBP1	Hs.644004
0.0059828	0.473	3.12	LUM	Hs.406475
0.0059835	0.473	0.78	ATP5O	Hs.409140
0.0059866	0.473	1.41	DRAM2	Hs.485606
0.0059982	0.473	1.48	GPR137B	Hs.498160
0.0060215	0.473	2.16	EBP	Hs.30619
0.0061933	0.473	0.64	LOC100506207	Hs.311049
0.0062156	0.473	0.49	TMC5	Hs.115838
0.0062479	0.473	1.33	DGKA	Hs.524488
0.0064748	0.473	1.41	SP4	Hs.88013
0.0064875	0.473	2.67	SFN	Hs.523718
0.0065013	0.473	0.61	RFTN2	Hs.591615
0.0066057	0.473	1.25	ZMYND8	Hs.446240
0.0066477	0.473	0.44	KCNQ3	Hs.374023
0.0068348	0.473	1.6	STK36	Hs.471404
0.0069343	0.473	0.61	PAX8	Hs.469728
0.0070268	0.473	0.55	ZNF273	Hs.520889
0.0072107	0.473	0.61	SEZ6L2	Hs.6314
0.0072125	0.473	0.66	RRAGC	Hs.532461
0.0072306	0.473	1.27	PWP1	Hs.506652
0.0072578	0.473	1.45	MMD	Hs.463483
0.0072933	0.473	1.56	INTS4	Hs.533723
0.0073012	0.473	0.53	UCP3	Hs.101337
0.0073522	0.473	1.64	ANKRD44	Hs.432706
0.0073812	0.473	0.57	LPCAT4	Hs.352614
0.0073846	0.473	1.36	B4GALT5	Hs.370487
0.0073885	0.473	1.53	HEATR2	Hs.535896
0.0073895	0.473	1.36	XPO6	Hs.460468

0.0075157	0.473	1.66	ZSCAN12	Hs.134816
0.0075613	0.473	2	TKT	Hs.89643
0.0076284	0.473	1.82	TMEM255A	Hs.437563
0.0077152	0.473	0.73	BCL7A	Hs.530970
0.0077686	0.473	0.56	MCFD2	Hs.662152
0.0078053	0.473	1.9	PIK3IP1	Hs.26670
0.0078941	0.473	2.94	TNFAIP3	Hs.211600
0.0079043	0.473	0.69	NLGN2	Hs.26229
0.0079922	0.473	0.72	ZSWIM3	Hs.292135
0.0080401	0.473	1.4	CD8A	Hs.85258
0.0080551	0.473	1.64	HMGB2	Hs.434953
0.008067	0.473	0.25	SERPINB13	Hs.241407
0.0081337	0.473	0.4	ITGA9	Hs.113157
0.0081797	0.473	1.98	S100A4	Hs.654444
0.0081901	0.473	1.69	LSS	Hs.596543
0.0082228	0.473	3.1	IGKC	Hs.449609
0.0082567	0.473	1.53	PRMT2	Hs.154163
0.0084426	0.473	1.45	CYR61	Hs.8867
0.0084669	0.473	0.72	SH3RF2	Hs.443728
0.0084831	0.473	1.88	NNMT	Hs.503911
0.0084925	0.473	1.86	FAT1	Hs.481371
0.00851	0.473	0.72	EDAR	Hs.171971
0.0086478	0.473	0.76	ELOVL5	Hs.743970
0.0086755	0.473	1.79	PAM	Hs.369430
0.0087317	0.473	0.51	PRB1	Hs.631726
0.008791	0.473	0.68	IL17RE	Hs.390823
0.0087911	0.473	0.55	ITGB3	Hs.218040
0.0089304	0.473	1.48	MNDA	Hs.153837
0.0089439	0.473	1.36	TMEM198	Hs.446664
0.0089917	0.473	2.01	CRISPLD2	Hs.513779
0.0090074	0.473	2.28	PIP4K2B	Hs.743962
0.0090574	0.473	0.8	KIAA1217	Hs.445885
0.009162	0.473	0.58	LOC100996793	Hs.741472
0.0091821	0.473	0.7	FAM108B1	Hs.380389
0.009232	0.473	1.33	KIF13A	Hs.94499
0.0092325	0.473	0.62	IQCE	Hs.520627

0.009338	0.473	0.79	TPT1-AS1	Hs.375766
0.0093719	0.473	1.21	PRDM2	Hs.371823
0.0093959	0.473	0.63	GGACT	Hs.350868
0.0094094	0.473	0.68	EFNA2	Hs.741510
0.0094703	0.473	1.85	GIMAP1	Hs.647079
0.0097491	0.473	1.52	RASGRP1	Hs.591127
0.0098425	0.473	2.3	TMOD2	Hs.513734
0.0098708	0.473	2.12	MSN	Hs.87752
0.009904	0.473	0.6	LIMK1	Hs.647035
0.0099327	0.473	0.64	COCH	Hs.21016
0.0099739	0.473	1.93	CAPN2	Hs.350899
0.0099987	0.473	0.56	SF3B2	Hs.406423
0.010018	0.473	1.45	ADCY7	Hs.513578
0.0100775	0.473	0.53	ADAMTS9-AS2	Hs.660881
0.0101254	0.473	1.45	HIGD2A	Hs.534575
0.0101441	0.473	1.32	EEF1D	Hs.333388
0.0102137	0.473	3.1	CHI3L1	Hs.382202
0.0102457	0.473	1.49	TCF4	Hs.605153
0.0103293	0.473	1.34	ZKSCAN3	Hs.380930
0.0103746	0.473	1.3	LURAP1L	Hs.445356
0.0103923	0.473	2.02	SRPX	Hs.15154
0.0104866	0.473	0.65	PAPOLA	Hs.253726
0.0105411	0.473	2.26	UBLCP1	Hs.591733
0.010566	0.473	1.21	E2F5	Hs.445758
0.0106079	0.473	1.84	LAPTM5	Hs.371021
0.0106115	0.473	2.1	ITPR1	Hs.567295
0.0106585	0.473	1.34	CLDN2	Hs.522746
0.0106792	0.473	1.27	DLG5	Hs.652690
0.0107435	0.473	0.55	ELAVL4	Hs.213050
0.0107508	0.473	1.37	OTUD1	Hs.499042
0.0107795	0.473	2.39	CD44	Hs.502328
0.0108328	0.473	1.32	NBAS	Hs.467759
0.0108438	0.473	1.6	YPEL3	Hs.513491
0.0108595	0.473	2.45	COL14A1	Hs.409662
0.0108927	0.473	1.71	RPGR	Hs.61438
0.0110173	0.473	0.62	CYGB	Hs.95120

0.0110452	0.473	1.67	SLC25A24	Hs.656870
0.0111025	0.473	0.77	PFDN6	Hs.446374
0.0111432	0.473	0.72	THAP6	Hs.479971
0.0111836	0.473	1.26	CD58	Hs.34341
0.0111907	0.473	0.36	WT1	Hs.591980
0.0112626	0.473	1.33	SYNPO2	Hs.655519
0.0113323	0.473	1.98	S100A10	Hs.143873
0.0113691	0.473	1.27	ZNF44	Hs.296731
0.0114204	0.473	2.1	COTL1	Hs.289092
0.0114714	0.473	0.7	PDPK1	Hs.459691
0.0114816	0.473	2.38	ADAM28	Hs.174030
0.0115177	0.473	1.55	RRAGD	Hs.31712
0.0115353	0.473	0.73	ZBTB10	Hs.591868
0.0115694	0.473	1.33	KIAA0930	Hs.592207
0.0116297	0.473	1.45	SUDS3	Hs.416630
0.0116542	0.473	1.25	GGA3	Hs.87726
0.0117248	0.473	0.71	METAP1D	Hs.298250
0.0117635	0.473	1.73	NIN	Hs.310429
0.0118215	0.473	0.6	ST6GAL2	Hs.98265
0.0118356	0.473	1.54	MCM3AP	Hs.389037
0.0118413	0.473	0.53	SMARCC1	Hs.476179
0.0119073	0.473	0.58	TTC18	Hs.591367
0.0119096	0.473	0.52	MAP6D1	Hs.478465
0.0119721	0.473	0.55	APBA2	Hs.618112
0.0119855	0.473	0.52	RASSF6	Hs.529677
0.0119966	0.473	0.77	RORC	Hs.256022
0.0120108	0.473	1.4	SLC2A13	Hs.558595
0.0120614	0.473	0.77	POLR3G	Hs.282387
0.0121235	0.473	0.61	MAP6	Hs.585540
0.0122006	0.473	1.31	LAMTOR4	Hs.406520
0.0122335	0.473	1.78	HSPG2	Hs.562227
0.0123315	0.473	1.37	ZRSR2	Hs.171909
0.012399	0.473	0.66	ABCD1	Hs.159546
0.012423	0.473	1.64	LOC158960	Hs.553131
0.0124806	0.473	1.47	ARHGEF7	Hs.508738
0.0124815	0.473	1.69	FAM117B	Hs.471130

0.0124836	0.473	2.01	ME1	Hs.21160
0.0125406	0.473	0.78	ZC3H7B	Hs.592188
0.0126178	0.473	2.85	DOCK8	Hs.132599
0.0126774	0.473	1.31	DENND1A	Hs.568340
0.01273	0.473	1.73	LCP1	Hs.381099
0.0127502	0.473	1.56	DSG2	Hs.412597
0.012755	0.473	1.66	C10orf118	Hs.159066
0.012799	0.473	0.64	ZNF782	Hs.732126
0.0128182	0.473	1.62	TRBC1	Hs.382212
0.0128331	0.473	0.79	MYO19	Hs.302051
0.0128813	0.473	1.73	LNPEP	Hs.527199
0.012962	0.473	0.74	LINC00202-1	Hs.529965
0.0129652	0.473	2.04	DPY19L1P1	Hs.633705
0.0130013	0.473	1.66	SETD4	Hs.606200
0.0130243	0.473	3.85	LOC374443	Hs.569124
0.0130907	0.473	1.6	ITGA4	Hs.440955
0.0131217	0.473	0.7	ZNF765	Hs.685640
0.0131338	0.473	1.35	RNPS1	Hs.355643
0.0131375	0.473	1.58	LOH12CR2	Hs.67553
0.0131536	0.473	0.71	HNF1A	Hs.654455
0.0133808	0.473	0.64	U2SURP	Hs.596572
0.0134254	0.473	0.69	PPA2	Hs.654957
0.0134699	0.473	0.79	CASC4	Hs.512867
0.0134716	0.473	2.5	TNFSF11	Hs.333791
0.0134742	0.473	0.78	CDK12	Hs.416108
0.0134931	0.473	0.49	MAGOHB	Hs.104650
0.0135668	0.473	0.62	MLLT10P1	Hs.653099
0.0135869	0.473	0.44	OLAH	Hs.24309
0.0136961	0.473	2.98	ABCD4	Hs.94395
0.0137177	0.473	1.41	IFNGR1	Hs.520414
0.0137674	0.473	1.27	PEG3	Hs.731875
0.0137857	0.473	1.6	C1orf162	Hs.288010
0.0137999	0.473	1.22	NCSTN	Hs.517249
0.013802	0.473	1.37	IRF8	Hs.137427
0.0138095	0.473	1.95	RAC2	Hs.517601
0.0138795	0.473	2.06	CARD10	Hs.57973

0.0138843	0.473	2.11	CXCL10	Hs.632586
0.0139486	0.473	0.67	JRK	Hs.535903
0.0139719	0.473	0.59	MCM3AP-AS1	Hs.655095
0.0140908	0.473	0.65	C2CD3	Hs.557938
0.0142058	0.473	1.48	SLC22A18	Hs.50868
0.0142866	0.473	0.57	MYO15A	Hs.462390
0.0143381	0.473	0.73	MAP3K13	Hs.591306
0.0143469	0.473	1.97	ECSCR	Hs.483538
0.0143835	0.473	0.69	LOC254100	Hs.595927
0.0143846	0.473	1.44	CCNY	Hs.14745
0.0144395	0.473	0.84	LOC221272	Hs.586272
0.0145295	0.473	1.32	NCAPD2	Hs.5719
0.0146358	0.473	0.67	TSC22D3	Hs.741199
0.0147317	0.473	0.59	NEU3	Hs.191074
0.0147815	0.473	1.39	SAMD9	Hs.65641
0.0147858	0.473	1.94	HLA-C	Hs.656020
0.0148449	0.473	0.61	MPDU1	Hs.246381
0.0148476	0.473	1.36	FOXRED1	Hs.317190
0.0148667	0.473	1.26	HIST3H2A	Hs.26331
0.0148868	0.473	1.58	TRIM6	Hs.729048
0.0149388	0.473	1.59	BBX	Hs.124366
0.0149416	0.473	1.49	CEP170	Hs.533635
0.014949	0.473	2.29	IQGAP1	Hs.430551
0.0149792	0.473	1.35	TUBA1B	Hs.524390
0.015032	0.473	1.33	CDK19	Hs.584867
0.015032	0.473	0.7	GATA4	Hs.243987
0.0150508	0.473	0.65	PPP1R37	Hs.285363
0.015071	0.473	0.74	ZC3H15	Hs.741180
0.0151372	0.473	0.77	PLA2G16	Hs.502775
0.0151501	0.473	1.52	IFT80	Hs.478095
0.015178	0.473	1.41	RAB22A	Hs.529044
0.0151812	0.473	1.22	CYTH3	Hs.487479
0.0152722	0.473	1.45	PNKD	Hs.98475
0.015296	0.473	0.37	DDX3Y	Hs.99120
0.0153091	0.473	1.24	POLG2	Hs.437009
0.0153129	0.473	1.27	PRDM11	Hs.178715

0.0153256	0.473	0.46	DST	Hs.604915
0.015328	0.473	1.56	ZNF682	Hs.306298
0.015339	0.473	0.81	FLJ36840	Hs.591832
0.0153932	0.473	1.4	TTL	Hs.358997
0.0155614	0.473	0.51	RAB11FIP4	Hs.406788
0.0156316	0.473	1.38	ZBTB2	Hs.520073
0.0156663	0.473	0.77	ATP11A	Hs.29189
0.0156828	0.473	0.4	DSC3	Hs.41690
0.0157087	0.473	1.69	TMEM234	Hs.272299
0.0157695	0.473	1.47	KCTD11	Hs.592112
0.0158498	0.473	0.59	FBXO9	Hs.216653
0.0158612	0.473	0.73	CCNJ	Hs.596479
0.0158671	0.473	1.97	CRIM1	Hs.699247
0.0159128	0.473	0.58	SCN8A	Hs.436550
0.0159679	0.473	1.69	COMMD2	Hs.432729
0.0159768	0.473	1.4	ACER3	Hs.23862
0.016065	0.473	1.53	AGTR1	Hs.477887
0.0161062	0.473	1.87	VIM	Hs.455493
0.0161355	0.473	3.43	CLEC7A	Hs.143929
0.0161735	0.473	0.75	PHF21A	Hs.502458
0.0161998	0.473	1.26	HIF1AN	Hs.500788
0.0162198	0.473	1.26	SOCS5	Hs.468426
0.0162318	0.473	0.49	C4orf36	Hs.339646
0.0162608	0.473	1.31	FYN	Hs.390567
0.0162669	0.473	1.39	SPATA20	Hs.103147
0.0163783	0.473	1.56	FUT4	Hs.390420
0.0164221	0.473	0.71	FLJ44342	Hs.710026
0.0165443	0.473	2.36	NAGK	Hs.7036
0.0165672	0.473	0.57	NKAIN2	Hs.656604
0.0165876	0.473	0.7	BRF1	Hs.424484
0.0166522	0.473	0.64	PDGFRB	Hs.509067
0.0167174	0.473	2.06	MAGEE1	Hs.8453
0.0167394	0.473	0.6	TCL6	Hs.510368
0.016768	0.473	2.17	HAVCR2	Hs.710500
0.0167868	0.473	1.47	CD244	Hs.157872
0.0168559	0.473	2.07	KAT6B	Hs.35758

0.0168633	0.473	2.33	TOP2A	Hs.156346
0.0168749	0.473	2.4	AFAP1	Hs.529369
0.0169058	0.473	0.58	CUX2	Hs.124953
0.016913	0.473	1.45	TCP11L2	Hs.696047
0.0169332	0.473	1.84	CLEC4A	Hs.504657
0.0169686	0.473	0.82	RPRD2	Hs.213666
0.0169914	0.473	1.62	APAF1	Hs.552567
0.0171316	0.473	1.42	TCF20	Hs.475018
0.0171395	0.473	1.73	ARSB	Hs.149103
0.0173189	0.473	1.16	TMEM11	Hs.592945
0.0173483	0.473	1.13	YTHDC1	Hs.175955
0.0173534	0.473	2.18	ZNF320	Hs.369632
0.0174156	0.473	1.95	LGALS2	Hs.531776
0.0174455	0.473	2.1	IGFBP7	Hs.479808
0.0174459	0.473	1.44	CCNA2	Hs.58974
0.0174612	0.473	0.5	C1orf173	Hs.531182
0.0175194	0.473	1.43	CKLF	Hs.15159
0.017559	0.473	2.42	HLA-DRA	Hs.520048
0.0175778	0.473	1.53	VOPP1	Hs.488307
0.0175854	0.473	1.23	USP46	Hs.7966
0.0176346	0.473	0.64	LOC100505676	Hs.121613
0.0177442	0.473	1.73	ZEB2	Hs.34871
0.0177446	0.473	1.93	FGL2	Hs.520989
0.0177457	0.473	0.56	ZNF568	Hs.404220
0.0178045	0.473	0.58	SPG20	Hs.440414
0.0178727	0.473	0.82	RNF216	Hs.487458
0.0178976	0.473	1.91	FZD1	Hs.94234
0.0179511	0.473	0.8	TLK1	Hs.596942
0.0179556	0.473	2.88	CPVL	Hs.233389
0.0179587	0.473	1.56	PSMD13	Hs.134688
0.0180056	0.473	0.82	CDC42	Hs.467637
0.0180449	0.473	1.45	FBXL21	Hs.591275
0.0181655	0.473	1.29	FBXL14	Hs.367956
0.0181716	0.473	2.43	JUNB	Hs.25292
0.0182332	0.473	0.5	MOG	Hs.141308
0.0182726	0.473	1.53	SPRY1	Hs.436944

0.0183526	0.473	2.55	DHCR7	Hs.503134
0.0183761	0.473	0.66	RFPL3	Hs.558455
0.0184037	0.473	1.37	SREK1IP1	Hs.69504
0.0184571	0.473	0.49	RET	Hs.350321
0.0184808	0.473	1.78	CCNG2	Hs.740456
0.0184933	0.473	0.69	BRCA1	Hs.194143
0.0185179	0.473	1.63	DPM3	Hs.110477
0.0185327	0.473	2.08	NCF2	Hs.587558
0.0185465	0.473	0.86	USP15	Hs.434951
0.0185534	0.473	2.76	MAP3K8	Hs.432453
0.018599	0.473	2.12	KCTD12	Hs.644125
0.0186525	0.473	0.71	AGFG1	Hs.352962
0.0186673	0.473	1.76	NEO1	Hs.388613
0.0186778	0.473	1.47	GLUD2	Hs.368538
0.0186924	0.473	1.54	ELTD1	Hs.132314
0.0187423	0.473	0.78	DHRS12	Hs.266728
0.0187973	0.473	1.83	ABHD12	Hs.441550
0.0188095	0.473	1.71	TLR2	Hs.519033
0.0188699	0.473	0.48	MROH1	Hs.443139
0.0188936	0.473	0.71	CBFA2T2	Hs.153934
0.0189088	0.473	0.77	CCDC176	Hs.644621
0.0189554	0.473	1.47	RAB11FIP1	Hs.191179
0.0190572	0.473	1.49	FAM65A	Hs.152717
0.0191258	0.473	1.39	PECAM1	Hs.376675
0.0191313	0.473	1.62	DKK3	Hs.292156
0.0191975	0.473	1.85	COMT	Hs.370408
0.0192209	0.473	2.12	NEU1	Hs.520037
0.0192642	0.473	0.67	TLE4	Hs.444213
0.0192706	0.473	0.58	ELP6	Hs.311100
0.0193324	0.473	1.8	CD14	Hs.163867
0.0193927	0.473	1.37	EIF1AX	Hs.522590
0.0194401	0.473	1.41	SYNJ2	Hs.434494
0.0194667	0.473	0.69	DNAJC30	Hs.647046
0.0195192	0.473	0.71	RIBC1	Hs.351743
0.0195679	0.473	1.41	KCNJ16	Hs.463985
0.0195697	0.473	2.19	CD74	Hs.436568

0.019641	0.473	1.32	DNTTIP1	Hs.472852
0.0196619	0.473	1.48	MPV17	Hs.75659
0.019693	0.473	1.44	EXT1	Hs.492618
0.0197006	0.473	3.38	LYZ	Hs.524579
0.0197809	0.473	0.79	EHMT1	Hs.495511
0.0198132	0.473	2.06	ZSWIM7	Hs.593985
0.0198874	0.473	1.44	ASXL2	Hs.119815
0.0199101	0.473	1.39	PRKD2	Hs.466987
0.0199165	0.473	1.55	CDKL3	Hs.719926
0.0199224	0.473	1.55	TNRC6B	Hs.372082
0.019931	0.473	0.54	CADM4	Hs.370984
0.0199425	0.473	1.44	HSPBAP1	Hs.29169
0.0199843	0.473	1.2	PTPN9	Hs.445775
0.0200973	0.473	0.72	UBXN1	Hs.351296
0.0201526	0.473	0.45	TMEM229B	Hs.509707
0.0201546	0.473	1.68	RASA2	Hs.655941
0.0201587	0.473	1.4	DOCK7	Hs.406156
0.0201635	0.473	0.68	SLC30A5	Hs.631975
0.0201729	0.473	0.65	NLN	Hs.247460
0.0201857	0.473	1.9	PI4KA	Hs.529438
0.0201999	0.473	0.72	SNX5	Hs.316890
0.0203494	0.473	0.64	ALDH1A2	Hs.643455
0.0203687	0.473	1.6	LOC153546	Hs.596513
0.0204411	0.473	1.47	CIITA	Hs.701991
0.0204721	0.473	1.56	ZSWIM6	Hs.650537
0.0205196	0.473	0.73	NDUFS4	Hs.528222
0.020532	0.473	0.67	SPN	Hs.632188
0.0205361	0.473	1.53	CCL2	Hs.303649
0.0206068	0.473	1.87	DPYSL2	Hs.593187
0.0207271	0.473	2.24	HLA-DPB1	Hs.485130
0.0207884	0.473	1.31	LOC286052	Hs.100691
0.0208074	0.473	1.44	TFE3	Hs.730740
0.0208172	0.473	1.56	CLEC2B	Hs.85201
0.0208286	0.473	0.83	JMJD1C-AS1	Hs.659950
0.0208974	0.473	1.36	FAM43A	Hs.435080
0.0209869	0.473	0.75	ZNF563	Hs.663510

0.0210201	0.473	1.45	EPB41L4A	Hs.584954
0.021033	0.473	0.5	SCD5	Hs.379191
0.0210564	0.473	0.72	FLJ31958	Hs.706372
0.0210663	0.473	0.76	LRRC8E	Hs.501511
0.0211428	0.473	1.61	ASAH1	Hs.527412
0.0211633	0.473	3.83	CXCL9	Hs.77367
0.0212078	0.473	2.62	CXCL14	Hs.483444
0.0212139	0.473	1.88	FUNDC1	Hs.7549
0.0212253	0.473	0.59	LOC340085	Hs.444279
0.0212801	0.473	1.71	IFI16	Hs.380250
0.0213016	0.473	0.81	MTRF1L	Hs.225836
0.0213272	0.473	1.28	PCSK7	Hs.648612
0.0214264	0.473	1.56	PDCL	Hs.271749
0.0215447	0.473	1.65	TUBA1C	Hs.652390
0.0215615	0.473	0.62	KCNJ15	Hs.411299
0.0215998	0.473	0.66	TCEB3	Hs.15535
0.0216029	0.473	0.66	COX3	Hs.591538
0.0216111	0.473	1.86	MARCKS	Hs.519909
0.0217038	0.473	1.38	HLA-E	Hs.650174
0.0217147	0.473	1.28	MAPKAP1	Hs.495138
0.0217237	0.473	0.65	RAET1E	Hs.511818
0.0217271	0.473	1.97	GBP1	Hs.62661
0.0217664	0.473	1.47	USP32P2	Hs.234573
0.0218757	0.473	2.26	SFRP1	Hs.213424
0.0219011	0.473	0.62	MOP-1	Hs.679418
0.021922	0.473	1.36	LZTFL1	Hs.174746
0.0219656	0.473	1.31	GNA13	Hs.515018
0.0219813	0.473	0.7	MAATS1	Hs.341906
0.0220501	0.473	1.62	TAF10	Hs.5158
0.0220896	0.473	0.65	NUPL1	Hs.507537
0.0222913	0.473	1.31	ADAMTS5	Hs.58324
0.0223662	0.473	0.83	RARB	Hs.654490
0.0223691	0.473	1.83	ZNF688	Hs.301463
0.0223955	0.473	1.58	RARRES1	Hs.131269
0.0224157	0.473	1.46	COG6	Hs.507805
0.0224408	0.473	0.77	SAA3P	Hs.454496

0.0225073	0.473	2.02	TRDMT1	Hs.351665
0.0225557	0.473	0.5	CXXC1P1	Hs.562045
0.0225694	0.473	0.71	MAN1A2	Hs.435938
0.0226163	0.473	1.21	LMBR1L	Hs.272838
0.0226353	0.473	1.87	TMSB10	Hs.446574
0.0227163	0.473	4.14	PQLC3	Hs.274415
0.022738	0.473	2.25	SERPINB1	Hs.381167
0.0227542	0.473	1.54	SEMA4D	Hs.494406
0.0228342	0.473	2.4	SKAP2	Hs.200770
0.0228455	0.473	0.54	FERMT1	Hs.472054
0.0229297	0.473	0.71	SLC34A3	Hs.432442
0.023013	0.473	1.41	PEG3-AS1	
0.0231197	0.473	0.61	LOC100287290	Hs.729204
0.0231378	0.473	1.37	MRPL52	Hs.355935
0.023158	0.473	1.41	TRAFD1	Hs.5148
0.0231598	0.473	2.03	ICAM1	Hs.643447
0.0231736	0.473	1.36	COL6A1	Hs.474053
0.0231747	0.473	1.58	THBS3	Hs.169875
0.0232499	0.473	1.42	FCGR2C	Hs.656750
0.0232509	0.473	1.72	HMGN4	Hs.236774
0.0233016	0.473	1.33	PLCG2	Hs.413111
0.0233248	0.473	1.92	PCOLCE	Hs.202097
0.0233312	0.473	0.59	FFAR1	Hs.248127
0.0234883	0.473	1.4	CCT7	Hs.368149
0.0235039	0.473	0.7	ELN	Hs.647061
0.0235066	0.473	0.76	CYB5D1	Hs.27475
0.0235331	0.473	2.2	ZNF468	Hs.467223
0.0235574	0.473	1.56	LOC100131541	Hs.661972
0.02363	0.473	2.23	MCTP2	Hs.33368
0.0237088	0.473	0.72	PRRC2C	Hs.494614
0.0237498	0.473	2.46	IKZF1	Hs.435949
0.0238191	0.473	0.79	SENP3	Hs.513926
0.0238479	0.473	0.68	CRYBB2P1	Hs.571835
0.0239229	0.473	0.53	GAFA1	Hs.661621
0.0239425	0.473	1.85	GOLGA8A	Hs.182982
0.0239451	0.473	1.46	AKAP7	Hs.486483

0.0239925	0.473	2.33	TAGAP	Hs.529984
0.0240011	0.473	1.63	TMEM97	Hs.199695
0.0240176	0.473	0.57	MLXIP	Hs.721711
0.0240982	0.473	1.7	FRMD6	Hs.434914
0.0241317	0.473	0.67	PLCXD2	Hs.477114
0.0241545	0.473	2.2	SCPEP1	Hs.514950
0.0241715	0.473	1.62	USP28	Hs.503891
0.0242684	0.473	0.24	SPOCK3	Hs.481133
0.0242729	0.473	0.7	MRPS27	Hs.482491
0.0242794	0.473	0.68	ASB14	Hs.665154
0.0243205	0.473	1.27	STK4	Hs.472838
0.024331	0.473	0.65	BCL2L14	Hs.210343
0.0243503	0.473	0.79	ATP5SL	Hs.744004
0.0243619	0.473	1.72	TXNIP	Hs.533977
0.0244086	0.473	0.7	TNPO1	Hs.482497
0.0244146	0.473	3.19	IL32	Hs.943
0.0244523	0.473	1.72	DAXX	Hs.336916
0.0244603	0.473	1.24	UBE2N	Hs.524630
0.0244753	0.473	1.68	ZNF558	Hs.659797
0.0244782	0.473	0.64	RECQL5	Hs.632229
0.0245	0.473	0.58	PSPN	Hs.248159
0.024638	0.473	0.72	TMEM259	Hs.515003
0.0246452	0.473	2.06	TMED9	Hs.279929
0.0246512	0.473	1.67	PPP1R18	Hs.101150
0.0246661	0.473	1.49	CEP112	Hs.408676
0.0247157	0.473	0.78	RREB1	Hs.298248
0.0247791	0.473	1.88	AEBP1	Hs.439463
0.02478	0.473	0.18	TXLNG2P	Hs.522863
0.0248229	0.473	1.91	SLC39A8	Hs.288034
0.0249625	0.473	0.64	TERF2	Hs.63335
0.0249651	0.473	1.39	SMUG1	Hs.632721
0.0250428	0.473	0.58	KIF25-AS1	Hs.225962
0.0251098	0.473	2.69	PHLDA1	Hs.602085
0.0252069	0.473	1.36	MYADM	Hs.380906
0.0252233	0.473	0.76	FAM227B	Hs.712959
0.0252714	0.473	2.22	CTSC	Hs.128065

0.0252772	0.473	0.68	SIRT6	Hs.423756
0.025337	0.473	1.51	HLA-F-AS1	Hs.646985
0.0253408	0.473	1.45	HARS2	Hs.432560
0.0254052	0.473	0.67	MIR4448	
0.0254562	0.473	0.86	POLH	Hs.655467
0.0255357	0.473	1.79	ATP9A	Hs.743248
0.0255369	0.473	1.2	DPCD	Hs.658128
0.0255843	0.473	1.68	ZFYVE1	Hs.335106
0.0255855	0.473	1.59	ARPC3	Hs.524741
0.0256191	0.473	0.56	LINC00487	Hs.446195
0.0256745	0.473	1.33	RBPJ	Hs.479396
0.0256806	0.473	0.67	CAMSAP1	Hs.522493
0.0257858	0.473	1.55	BCL2	Hs.150749
0.0258132	0.473	0.73	GPATCH2	Hs.420757
0.0258764	0.473	1.95	APOE	Hs.654439
0.0259005	0.473	1.23	SEPT6	Hs.496666
0.0259079	0.473	1.65	IFITM2	Hs.709321
0.0259219	0.473	1.53	TIPRL	Hs.209431
0.0259328	0.473	0.73	ERVK13-1	Hs.406976
0.0259595	0.473	1.35	GPATCH2L	Hs.410231
0.0259628	0.473	1.83	MAST3	Hs.466184
0.0260556	0.473	2.05	MAP1LC3B	Hs.356061
0.0260716	0.473	0.73	THADA	Hs.369592
0.0261293	0.473	0.11	KDM5D	Hs.80358
0.0262177	0.473	0.67	PTPRK	Hs.155919
0.0262559	0.473	1.36	HIVEP1	Hs.567284
0.0262861	0.473	0.65	PRINS	Hs.731140
0.0262882	0.473	1.25	MAPKAPK5-AS1	Hs.333120
0.0263145	0.473	2.5	GAS2L3	Hs.20575
0.0263191	0.473	1.49	DACH1	Hs.129452
0.0263777	0.473	0.53	LOC100506343	Hs.485810
0.0264052	0.473	0.73	FBXO42	Hs.522384
0.0265002	0.473	1.79	CLEC2D	Hs.268326
0.0265088	0.473	1.64	MYD88	Hs.82116
0.026553	0.473	0.8	MTMR7	Hs.625674
0.0266163	0.473	0.82	PCMTD1	Hs.671268

0.026652	0.473	1.41	SEMA6A	Hs.156967
0.0267528	0.473	0.85	PRPF18	Hs.729158
0.0267686	0.473	1.42	C6orf47	Hs.247323
0.0269395	0.473	0.72	TUBGCP6	Hs.336431
0.0269708	0.473	1.36	NHP2L1	Hs.182255
0.0269769	0.473	1.91	SLC15A4	Hs.507260
0.0269839	0.473	0.7	CYP2U1	Hs.109087
0.0269986	0.473	1.45	SWAP70	Hs.153026
0.0270019	0.473	1.36	FGD6	Hs.506381
0.0270163	0.473	1.2	KRT8	Hs.533782
0.0270254	0.473	1.51	NOP16	Hs.696283
0.02705	0.473	0.79	ATF7IP	Hs.591151
0.0270827	0.473	0.86	AGAP9	Hs.645346
0.0271068	0.473	1.68	RNF38	Hs.333503
0.0271435	0.473	0.51	OMG	Hs.113874
0.0271538	0.473	1.48	SLC30A9	Hs.479634
0.0271645	0.473	1.42	GNPTG	Hs.241575
0.0271646	0.473	0.57	VSX1	Hs.274264
0.0271877	0.473	0.78	SCAF4	Hs.17255
0.0272238	0.473	1.57	CYP4X1	Hs.439760
0.0272885	0.473	1.6	MRPL23	Hs.3254
0.0273732	0.473	0.43	SCN11A	Hs.591657
0.027391	0.473	1.76	QKI	Hs.510324
0.027402	0.473	1.31	PLXND1	Hs.301685
0.0274279	0.473	2.36	C10orf10	Hs.93675
0.0274859	0.473	0.82	NT5C3	Hs.487933
0.0275677	0.473	0.78	VPS36	Hs.109520
0.0276154	0.473	1.99	ELMO2	Hs.210469
0.0276279	0.473	0.72	EFCAB13	Hs.463303
0.0276306	0.473	1.32	NTMT1	Hs.744027
0.0276889	0.473	0.77	FAM206A	Hs.29276
0.0276919	0.473	0.64	NKAIN3	Hs.654662
0.0277072	0.473	0.76	ZNF540	Hs.728927
0.0277362	0.473	0.61	CCDC38	Hs.210377
0.0278315	0.473	0.77	ANAPC16	Hs.426296
0.027887	0.473	1.73	ANXA1	Hs.494173

0.0279059	0.473	1.75	CD83	Hs.595133
0.0279821	0.473	1.62	CDC42SE2	Hs.508829
0.0280548	0.473	0.62	MCL1	Hs.632486
0.0280589	0.473	1.96	HCK	Hs.655210
0.0280984	0.473	0.74	GATAD2B	Hs.4779
0.0281468	0.473	2.24	PTPRC	Hs.654514
0.028159	0.473	1.71	WIPF1	Hs.128067
0.0281756	0.473	1.34	CYB5R4	Hs.5741
0.0281914	0.473	1.32	ZBTB11	Hs.655286
0.0282452	0.473	0.8	SRRM2	Hs.433343
0.0282592	0.473	0.85	ZNF142	Hs.657969
0.0282948	0.473	0.87	POLR3E	Hs.460298
0.0282986	0.473	0.63	DPEP3	Hs.302028
0.0283009	0.473	0.78	LINC00630	Hs.687084
0.0283034	0.473	1.38	RCAN3	Hs.656799
0.0283688	0.473	1.43	PLXDC2	Hs.658134
0.0283857	0.473	0.53	LOC100288310	Hs.720615
0.0284108	0.473	2.62	MXRA5	Hs.369422
0.0284223	0.473	1.44	TMEM65	Hs.187646
0.0284347	0.473	1.34	CNOT4	Hs.490224
0.0285033	0.473	1.53	GNG11	Hs.83381
0.0285854	0.473	0.53	GABRR2	Hs.719891
0.0286848	0.473	1.37	RCSD1	Hs.493867
0.0287022	0.473	1.79	TNFAIP8	Hs.656274
0.028716	0.473	1.8	DDX28	Hs.458313
0.0287496	0.473	1.34	UBA1	Hs.533273
0.0287804	0.473	2.38	ITGAM	Hs.172631
0.0287875	0.473	1.82	TMPRSS6	Hs.370885
0.0288177	0.473	1.23	RAB5B	Hs.157659
0.0288341	0.473	1.61	SPTLC2	Hs.435661
0.0288902	0.473	0.79	EZH1	Hs.194669
0.028895	0.473	1.57	ANTXR1	Hs.165859
0.0288952	0.473	1.19	DVL2	Hs.118640
0.0289055	0.473	0.47	LOC100292680	Hs.391695
0.0289097	0.473	0.78	CHAD	Hs.97220
0.0289642	0.473	1.61	FYB	Hs.370503

0.0289765	0.473	0.69	IGFBP5	Hs.607212
0.0289831	0.473	0.66	GLRX3	Hs.42644
0.0289935	0.473	0.76	WDR74	Hs.654620
0.0290809	0.473	0.44	LOC100506675	Hs.446169
0.0290984	0.473	0.77	SLC16A6	Hs.42645
0.0291295	0.473	1.61	ETV5	Hs.43697
0.0291327	0.473	1.22	PLXNB1	Hs.476209
0.029162	0.473	1.65	AP1S2	Hs.653504
0.0292257	0.473	0.72	ZNF641	Hs.23492
0.0292373	0.473	2.08	GIMAP7	Hs.647074
0.029252	0.473	1.61	STS	Hs.522578
0.0292576	0.473	2.39	TIMP2	Hs.633514
0.0292826	0.473	1.45	MAF	Hs.134859
0.0292944	0.473	1.33	TRAPPC2L	Hs.461722
0.0293025	0.473	1.86	THOC2	Hs.149991
0.0293086	0.473	0.86	PA2G4	Hs.524498
0.0293411	0.473	0.74	LOC100134822	Hs.518952
0.0294209	0.473	1.58	PDGFRA	Hs.74615
0.029515	0.473	2.69	TNFSF15	Hs.23349
0.0295371	0.473	1.37	STMN1	Hs.209983
0.0296366	0.473	0.67	LOC100505839	Hs.655149
0.0296438	0.473	1.48	DCBLD2	Hs.203691
0.0296929	0.473	1.33	BUD13	Hs.437341
0.0297035	0.473	1.73	IFI30	Hs.14623
0.0297073	0.473	0.73	ZNF7	Hs.493218
0.0297225	0.473	0.6	KAZN	Hs.368823
0.0297692	0.473	2.05	CXCL12	Hs.522891
0.0298793	0.473	1.61	LITAF	Hs.459940
0.0299227	0.473	1.96	CYBB	Hs.292356
0.0299533	0.473	2.06	NPC2	Hs.433222
0.0300046	0.473	0.82	MAP3K2	Hs.744145
0.0300284	0.473	0.64	HECTD4	Hs.530943
0.0300342	0.473	1.88	PMP22	Hs.372031
0.0300781	0.473	1.79	GSTM3	Hs.2006
0.0300792	0.473	0.81	PLAA	Hs.27182
0.0301069	0.473	1.58	ATM	Hs.367437

0.0301648	0.473	0.7	PARP11	Hs.657268
0.0301941	0.473	1.34	TMTC4	Hs.190983
0.0302516	0.473	1.64	FAM192A	Hs.396740
0.0302666	0.473	1.55	LEPROTL1	Hs.146585
0.0302776	0.473	1.43	FOXK1	Hs.487393
0.0303878	0.473	1.34	KIAA1432	Hs.211520
0.0304218	0.473	1.7	GPI	Hs.466471
0.0304367	0.473	2.11	TNFRSF11B	Hs.81791
0.0304587	0.473	1.38	ZKSCAN4	Hs.44720
0.0304676	0.473	0.51	HGS	Hs.661056
0.0305094	0.473	1.25	EME2	Hs.7247
0.0305111	0.473	0.62	FLJ32955	Hs.470217
0.0305132	0.473	1.54	LRBA	Hs.480938
0.0305862	0.473	0.69	BCR	Hs.517461
0.0306167	0.473	0.79	ZBED4	Hs.475208
0.0306335	0.473	2.03	PXDC1	Hs.484500
0.0306576	0.473	1.73	HLA-DOA	Hs.631991
0.0307244	0.473	0.73	TRMT44	Hs.566191
0.0307366	0.473	0.66	UBA6	Hs.212774
0.0307978	0.473	0.74	HBS1L	Hs.378532
0.0308027	0.473	0.51	KRBOX1	Hs.720172
0.0308629	0.473	1.96	DCN	Hs.156316
0.0310561	0.473	2	CD24	Hs.644105
0.0310972	0.473	1.66	MAFF	Hs.517617
0.0311652	0.473	0.6	SRSF7	Hs.309090
0.0311942	0.473	0.79	FAF1	Hs.530402
0.0312084	0.473	0.69	ALPK1	Hs.652825
0.0312119	0.473	1.4	DRAM1	Hs.525634
0.0312616	0.473	1.51	TDRD7	Hs.193842
0.0312662	0.473	1.89	FDPS	Hs.335918
0.0312775	0.473	2.59	HLA-DQB1	Hs.409934
0.0313092	0.473	0.7	LOC541473	Hs.695009
0.0313807	0.473	0.77	SP3	Hs.531587
0.0313945	0.473	1.77	SPATA2	Hs.48513
0.0314177	0.473	1.27	MRPL1	Hs.532019
0.0314554	0.473	0.63	LOC283914	Hs.385772

0.0315098	0.473	1.32	PHF20	Hs.517044
0.0315379	0.473	1.63	ARRB1	Hs.503284
0.0315428	0.473	0.61	NRGN	Hs.524116
0.031543	0.473	0.66	ARID3A	Hs.501296
0.0315785	0.473	1.41	RAPGEF2	Hs.113912
0.0315866	0.473	0.57	SERPINB10	Hs.158339
0.0316232	0.473	0.7	ELMOD2	Hs.450105
0.0316489	0.473	1.53	AGTPBP1	Hs.719980
0.0316694	0.473	1.8	TFEC	Hs.125962
0.0316805	0.473	1.37	ZBTB38	Hs.518301
0.0316836	0.473	2.74	SCD	Hs.558396
0.0317152	0.473	1.51	NCK2	Hs.529244
0.031716	0.473	1.33	LOC100506844	Hs.90286
0.0318055	0.473	0.66	TRIM65	Hs.189823
0.0318186	0.473	0.68	DNAH10	Hs.622654
0.0318259	0.473	1.88	INHBB	Hs.1735
0.0318334	0.473	1.39	ZBTB47	Hs.409561
0.0319038	0.473	0.59	STX6	Hs.518417
0.0319089	0.473	1.27	DTD1	Hs.659442
0.0319102	0.473	1.65	NREP	Hs.36053
0.0319467	0.473	1.66	TMEM165	Hs.479766
0.0319819	0.473	1.36	GLA	Hs.69089
0.0319891	0.473	2.18	FCGR2B	Hs.654395
0.0319927	0.473	1.52	SLC9A6	Hs.62185
0.032033	0.473	1.97	DPF2	Hs.13495
0.032046	0.473	1.66	TMEM57	Hs.189782
0.0320641	0.473	1.54	STXBP1	Hs.288229
0.0320685	0.473	0.84	TSEN15	Hs.548197
0.0320772	0.473	1.56	HLA-G	Hs.512152
0.0321614	0.473	0.34	LOC284561	Hs.504540
0.0321754	0.473	2.26	NLRC5	Hs.528836
0.0321793	0.473	0.47	NEGR1	Hs.146542
0.0322595	0.473	2.05	HLA-F	Hs.519972
0.0322781	0.473	0.46	AGMAT	Hs.461532
0.0323114	0.473	1.3	MED20	Hs.278434
0.0323224	0.473	0.83	RBM15B	Hs.118738

0.0323669	0.473	1.29	SIK2	Hs.269128
0.0324011	0.473	0.67	DPYSL4	Hs.100058
0.0324433	0.473	1.41	RPL15	Hs.381219
0.0324654	0.473	0.73	PCBP2	Hs.546271
0.0324703	0.473	1.86	PNMA1	Hs.194709
0.0325383	0.473	0.18	UTY	Hs.115277
0.0325579	0.473	1.84	CHD4	Hs.162233
0.03256	0.473	0.72	SMCR8	Hs.592944
0.0326451	0.473	1.47	LOC100505812	Hs.614136
0.0326684	0.473	1.82	ACSS2	Hs.517034
0.0327506	0.473	1.42	USP32	Hs.744129
0.0327852	0.473	1.57	PREX1	Hs.744064
0.0328106	0.473	0.54	TMEM174	Hs.508588
0.0328815	0.473	0.74	ERLIN2	Hs.705490
0.0328883	0.473	1.42	C11orf73	Hs.283322
0.0328894	0.473	1.6	MYO10	Hs.481720
0.03292	0.473	0.67	TCTN2	Hs.167165
0.0329292	0.473	1.33	TMEM48	Hs.476525
0.0329337	0.473	0.13	USP9Y	Hs.598540
0.0329632	0.473	1.73	SFXN3	Hs.283844
0.0329696	0.473	0.86	SIRT5	Hs.567431
0.0329812	0.473	0.73	AKD1	Hs.205144
0.0329835	0.473	1.25	SP110	Hs.145150
0.0330103	0.473	1.4	CLIC1	Hs.414565
0.0331027	0.473	0.54	SLC17A7	Hs.375616
0.0331075	0.473	1.47	ZNF235	Hs.724032
0.03317	0.473	0.71	DDHD1	Hs.125525
0.0331791	0.473	1.86	TLR8	Hs.660543
0.0331966	0.473	0.71	EPS15L1	Hs.654639
0.0332008	0.473	2.23	ACAT2	Hs.571037
0.0333098	0.473	2.16	POSTN	Hs.136348
0.0333113	0.473	1.32	FAT4	Hs.563205
0.0333209	0.473	1.9	MPEG1	Hs.709439
0.0333317	0.473	2.67	SLC7A7	Hs.513147
0.0333368	0.473	1.24	SMIM7	Hs.356467
0.03334	0.473	0.61	ITGB4	Hs.632226

0.0333461	0.473	1.5	HLA-DMA	Hs.728759
0.0333669	0.473	2.06	BCO2	Hs.728325
0.0334757	0.473	1.61	ITFG1	Hs.42217
0.0335415	0.473	1.87	NUP62	Hs.574492
0.0336998	0.473	0.88	KANSL1	Hs.648744
0.0337153	0.473	1.52	TMEM254	Hs.169982
0.033743	0.473	1.67	BIRC5	Hs.514527
0.0338632	0.473	1.45	CYSTM1	Hs.529798
0.0338821	0.473	0.79	PI4KB	Hs.632465
0.0339034	0.473	1.54	COG3	Hs.507948
0.0339157	0.473	1.18	TMOD3	Hs.4998
0.033938	0.473	1.36	TIMM50	Hs.590956
0.0340407	0.473	0.79	UBE2G2	Hs.529420
0.0340781	0.473	1.61	FCER1G	Hs.433300
0.0340863	0.473	0.58	RAB40C	Hs.459630
0.0341447	0.473	1.64	SEC22C	Hs.445892
0.034182	0.473	1.28	MAP3K1	Hs.653654
0.0342714	0.473	1.54	PTGDR	Hs.306831
0.0343059	0.473	1.43	FAM65B	Hs.559459
0.0343063	0.473	0.52	EPB41L1	Hs.743243
0.0343064	0.473	0.85	ZNF490	Hs.655860
0.0343221	0.473	1.22	ZCCHC17	Hs.524094
0.0343592	0.473	1.48	SPINT2	Hs.31439
0.0343768	0.473	1.29	MIEN1	Hs.333526
0.0343791	0.473	1.83	ITGBL1	Hs.696554
0.0344155	0.473	1.47	MARK3	Hs.35828
0.0344332	0.473	1.44	PDXDC1	Hs.370781
0.0344547	0.473	1.39	LOC550643	Hs.355559
0.0344911	0.473	0.74	C5orf22	Hs.519246
0.0344997	0.473	1.3	NF1	Hs.113577
0.0345196	0.473	1.26	KDM5B	Hs.443650
0.0345518	0.473	0.63	DES	Hs.594952
0.0345542	0.473	1.73	NEBL	Hs.5025
0.0345647	0.473	1.48	C16orf62	Hs.654964
0.034652	0.473	2.69	SLAMF7	Hs.517265
0.0346556	0.473	1.9	GLG1	Hs.109731

0.0346885	0.473	0.79	APOPT1	Hs.598441
0.0347149	0.473	1.33	SEPW1	Hs.631549
0.0347207	0.473	1.66	MRPL44	Hs.203559
0.0347913	0.473	1.62	SLC38A1	Hs.533770
0.034805	0.473	1.89	BIRC3	Hs.127799
0.0348066	0.473	2.25	OLFM1	Hs.522484
0.034815	0.473	1.89	LOC100996419	Hs.314414
0.0348701	0.473	0.8	WDTC1	Hs.469154
0.0348708	0.473	1.5	VPS35	Hs.454528
0.0348735	0.473	0.61	RECK	Hs.744121
0.0348836	0.473	1.9	MECR	Hs.183646
0.0348947	0.473	1.9	RIPK4	Hs.517310
0.0349034	0.473	1.89	FLII	Hs.513984
0.0349445	0.473	1.46	SLC2A3	Hs.419240
0.0349741	0.473	1.24	MACF1	Hs.472475
0.0349748	0.473	2.02	FOLR2	Hs.433159
0.0349992	0.473	0.65	PRKDC	Hs.491682
0.0350024	0.473	1.31	ITGAL	Hs.174103
0.0350353	0.473	1.28	RBP7	Hs.422688
0.0350471	0.473	0.44	CA1	Hs.23118
0.0350617	0.473	2.78	AJUBA	Hs.655832
0.0350774	0.473	1.54	FOXP2	Hs.282787
0.0351482	0.473	1.76	APRT	Hs.28914
0.035174	0.473	1.85	SOBP	Hs.445244
0.035196	0.473	1.61	MYL12A	Hs.190086
0.0352262	0.473	1.46	LRCH3	Hs.518414
0.035253	0.473	1.35	GUCY1B3	Hs.77890
0.0352595	0.473	1.61	CD84	Hs.398093
0.0352746	0.473	1.7	ZNHIT1	Hs.211079
0.0352924	0.473	1.68	TMEM39A	Hs.594171
0.035315	0.473	1.28	SMARCAL1	Hs.516674
0.0353252	0.473	1.36	AP1AR	Hs.435991
0.035382	0.473	2.6	GPNMB	Hs.190495
0.0353877	0.473	2.07	TMEM100	Hs.173233
0.0354307	0.473	0.73	HECTD2	Hs.596096
0.0354474	0.473	1.68	BTN3A1	Hs.191510

0.0354903	0.473	2.21	GTF3C4	Hs.656646
0.0355136	0.473	1.52	RPL26L1	Hs.546390
0.0355756	0.473	0.71	TGM2	Hs.517033
0.0355889	0.473	1.58	SEC14L1	Hs.464184
0.0356311	0.473	2.08	XAF1	Hs.441975
0.0356922	0.473	0.81	POFUT1	Hs.472409
0.0357365	0.473	0.82	RBM4	Hs.523822
0.0357552	0.473	1.75	ALKBH7	Hs.111099
0.0357572	0.473	2.34	HPGD	Hs.596913
0.0357762	0.473	0.69	WNK3	Hs.92423
0.0357927	0.473	2.09	DNPEP	Hs.258551
0.0358547	0.473	0.66	FRG1B	Hs.653099
0.0358604	0.473	1.38	SCRN1	Hs.520740
0.0358761	0.473	1.32	SPTAN1	Hs.372331
0.0359117	0.473	1.36	EIF6	Hs.743992
0.0359167	0.473	1.6	DOCK11	Hs.368203
0.0359512	0.473	1.82	A1BG	Hs.529161
0.0359561	0.473	0.58	CACTIN	Hs.128425
0.0359989	0.473	0.61	HERC2P4	Hs.531536
0.0360222	0.473	1.72	AHSA2	Hs.655602
0.0360368	0.473	0.81	SLC25A16	Hs.180408
0.036103	0.473	0.65	C3orf62	Hs.403828
0.0361573	0.473	1.56	ANKLE2	Hs.654628
0.0362455	0.473	1.38	MORN2	Hs.729207
0.0362658	0.473	0.38	WIF1	Hs.284122
0.036291	0.473	0.5	DCLK3	Hs.631907
0.036297	0.473	2.27	TFPI2	Hs.438231
0.0363715	0.473	1.3	ZWILCH	Hs.21331
0.0364095	0.473	1.31	SPATA7	Hs.525518
0.0364123	0.473	2.31	CECR1	Hs.170310
0.0364571	0.473	1.35	TMEM218	Hs.731899
0.0364878	0.473	0.78	ERVW-1	
0.0364904	0.473	1.72	GBP3	Hs.720167
0.0365307	0.473	0.64	PHLDA2	Hs.154036
0.0365347	0.473	1.7	SSR3	Hs.518346
0.0366083	0.473	0.52	CXorf58	Hs.351265

0.0366181	0.473	1.78	APLNR	Hs.438311
0.0367008	0.473	0.66	LOC100505738	Hs.42197
0.0367201	0.473	0.5	LOC283788	Hs.657131
0.0367371	0.473	1.74	GPR124	Hs.274136
0.0367412	0.473	1.48	BNIP3L	Hs.131226
0.0367449	0.473	1.48	PTGIS	Hs.302085
0.0367593	0.473	1.36	CSNK1G1	Hs.646508
0.0368297	0.473	1.5	PRPF38B	Hs.342307
0.0369196	0.473	1.46	SMA4	Hs.582500
0.036947	0.473	0.79	CHD2	Hs.220864
0.036961	0.473	1.28	PHACTR2	Hs.102471
0.0370046	0.473	1.53	RACGAP1	Hs.505469
0.0370076	0.473	2.14	COL3A1	Hs.443625
0.0370195	0.473	1.58	MICALL1	Hs.517610
0.0371921	0.473	0.66	RAP1GDS1	Hs.132858
0.037226	0.473	0.65	BCAM	Hs.625725
0.0372502	0.473	1.56	GTPBP2	Hs.485449
0.0372517	0.473	2.04	PRR15L	Hs.368260
0.0373059	0.473	0.64	MAB21L2	Hs.584852
0.0373756	0.473	0.77	PRPF4	Hs.744014
0.0373933	0.473	0.59	SYN3	Hs.608750
0.037415	0.473	1.86	CFTR	Hs.489786
0.0374419	0.473	1.36	AFTPH	Hs.655167
0.0374765	0.473	0.66	PDLIM7	Hs.533040
0.0375289	0.473	1.41	NBPF1	Hs.467587
0.0375706	0.473	0.7	LOC728705	Hs.129828
0.0375846	0.473	1.32	GTPBP8	Hs.127496
0.0376541	0.473	1.76	TPPP	Hs.481466
0.0376805	0.473	0.71	LOC100129961	Hs.729028
0.0376888	0.473	0.6	CPSF2	Hs.657632
0.0377831	0.473	1.51	ZNF555	Hs.47712
0.037785	0.473	1.68	GFPT1	Hs.580300
0.0377972	0.473	1.33	RAB42	Hs.652321
0.0377987	0.473	1.5	LOC692247	Hs.560022
0.0378603	0.473	1.37	MYO7A	Hs.370421
0.0379072	0.473	0.62	TCOF1	Hs.519672

0.0379211	0.473	1.79	TAGLN2	Hs.517168
0.0379278	0.473	0.74	ZNF551	Hs.109540
0.037957	0.473	0.72	TMEM135	Hs.188591
0.0379643	0.473	1.32	TMEM19	Hs.644802
0.0380225	0.473	0.74	PHC2	Hs.524271
0.0380486	0.473	2.07	HMBOX1	Hs.591836
0.0381134	0.473	2.52	HMGCR	Hs.628096
0.0381238	0.473	1.78	SPARC	Hs.111779
0.0381355	0.473	1.7	PLEKHA2	Hs.369123
0.0381391	0.473	0.56	KCNJ12	Hs.200629
0.0382073	0.473	2.04	ARL4C	Hs.111554
0.038209	0.473	0.76	C1QTNF1	Hs.201398
0.0383198	0.473	2.46	TBXA2R	Hs.442530
0.0384189	0.473	2.39	FDFT1	Hs.593928
0.0385034	0.473	1.28	TPP1	Hs.523454
0.0385225	0.473	2.09	HNRPLL	Hs.445497
0.0385843	0.473	0.66	ANKLE1	Hs.721610
0.0386419	0.473	0.82	DIAPH1	Hs.529451
0.0386427	0.473	2.01	KIF2A	Hs.558351
0.0386526	0.473	1.7	PIGR	Hs.497589
0.0386845	0.473	1.44	FLRT3	Hs.41296
0.0386916	0.473	1.37	M6PR	Hs.134084
0.038695	0.473	1.7	SMC4	Hs.58992
0.0387288	0.473	1.42	MIS18BP1	Hs.732769
0.0387441	0.473	0.55	C11orf58	Hs.645792
0.0388064	0.473	1.21	SET	Hs.436687
0.038811	0.473	0.62	CALB1	Hs.65425
0.0388681	0.473	1.2	RPP25L	Hs.15961
0.0388725	0.473	0.7	MACROD2-AS1	Hs.407533
0.0388781	0.473	1.65	SCN9A	Hs.439145
0.0388826	0.473	1.84	MDFIC	Hs.741413
0.0389248	0.473	1.69	FLI1	Hs.504281
0.0390218	0.473	0.58	CCDC11	Hs.658630
0.03925	0.473	0.7	PI4K2A	Hs.25300
0.0392575	0.473	0.76	ZNF268	Hs.124047
0.0392758	0.473	1.62	C1orf198	Hs.520494

0.0393527	0.473	0.85	PWWP2A	Hs.438851
0.0393675	0.473	0.67	MARCH6	Hs.432862
0.0394224	0.473	2.03	PELO	Hs.644352
0.0394466	0.473	1.67	SLC7A6	Hs.679580
0.0394476	0.473	1.26	ILF3	Hs.465885
0.0394562	0.473	1.39	PIH1D1	Hs.5245
0.0394713	0.473	1.54	FTO	Hs.528833
0.0395288	0.473	1.3	JAK2	Hs.656213
0.0395669	0.473	0.5	ATP13A5	Hs.658032
0.0395806	0.473	0.79	CDK5RAP2	Hs.269560
0.0395821	0.473	1.38	SRBD1	Hs.14229
0.0396076	0.473	1.59	HCLS1	Hs.14601
0.0396239	0.473	1.41	MPZL2	Hs.116651
0.0396444	0.473	1.3	TM7SF2	Hs.31130
0.0396567	0.473	1.49	GUSBP11	Hs.148656
0.0396678	0.473	1.66	SOCS2	Hs.485572
0.039672	0.473	1.22	KLHL18	Hs.517946
0.0396728	0.473	1.73	PASK	Hs.397891
0.039704	0.473	1.6	FAM102B	Hs.200230
0.0397219	0.473	0.6	ITSN1	Hs.160324
0.0397257	0.473	1.62	OAZ1	Hs.446427
0.0397851	0.473	1.77	ZFP90	Hs.461074
0.039794	0.473	1.36	DENND2D	Hs.557850
0.0398027	0.473	0.67	AUTS2	Hs.21631
0.0398261	0.473	1.3	ZBTB22	Hs.206770
0.0398575	0.473	1.71	MPZL1	Hs.493919
0.0398625	0.473	0.68	AKT3	Hs.498292
0.0398742	0.473	2.1	SSR4	Hs.409223
0.0398755	0.473	1.99	LYST	Hs.532411
0.0398932	0.473	1.43	CDKAL1	Hs.657604
0.0399212	0.473	1.58	NT5E	Hs.153952
0.0399772	0.473	1.49	CMPK2	Hs.7155
0.039986	0.473	1.67	WDR45	Hs.632807
0.0400271	0.473	1.6	DCHS1	Hs.199850
0.0400768	0.473	1.93	ZBTB33	Hs.143604
0.0400902	0.473	2.09	IGFBP3	Hs.450230

0.0400966	0.473	1.78	DBN1	Hs.130316
0.0401124	0.473	1.73	TRAPPC2	Hs.592238
0.0401248	0.473	1.8	ZAK	Hs.444451
0.0401724	0.473	1.45	DENND4A	Hs.654567
0.0401832	0.473	1.37	SLC30A4	Hs.162989
0.0401855	0.473	1.78	ANK3	Hs.499725
0.0402559	0.473	0.7	MGC12488	Hs.659351
0.0402567	0.473	1.41	SH3KBP1	Hs.726365
0.0402594	0.473	0.82	CAMSAP3	Hs.17686
0.0402668	0.473	1.32	YARS2	Hs.505231
0.0402987	0.473	1.34	HYI	Hs.709864
0.0403281	0.473	1.63	COL5A1	Hs.210283
0.0403917	0.473	1.33	ESPL1	Hs.153479
0.0403997	0.473	1.55	ZNF623	Hs.43133
0.0404138	0.473	1.33	PLOD1	Hs.75093
0.0404167	0.473	1.35	FAM204A	Hs.372309
0.0404434	0.473	0.58	BPNT1	Hs.406134
0.0404486	0.473	1.91	TMEM67	Hs.116240
0.0404547	0.473	1.25	TMEM242	Hs.157212
0.0404588	0.473	0.64	ZBED3-AS1	Hs.161338
0.0404754	0.473	0.66	LRP1	Hs.162757
0.0404947	0.473	0.7	MARCH3	Hs.132441
0.0405167	0.473	1.88	PTGFRN	Hs.418093
0.040538	0.473	1.72	TAOK1	Hs.597434
0.0405609	0.473	2.44	CD300A	Hs.9688
0.0405696	0.473	1.77	RBPMS2	Hs.436518
0.0405783	0.473	0.71	ADCK3	Hs.118241
0.0405928	0.473	0.77	LOC642533	Hs.54713
0.0406647	0.473	1.62	SESN3	Hs.120633
0.0406917	0.473	1.77	ZNF217	Hs.155040
0.0407459	0.473	1.64	RHPN2	Hs.466435
0.0407973	0.473	1.71	RDH10	Hs.244940
0.0408237	0.473	1.68	PTPN14	Hs.193557
0.0408278	0.473	1.28	PIAS2	Hs.657844
0.0408327	0.473	0.77	C5orf56	Hs.658288
0.0408446	0.473	0.85	ST3GAL3	Hs.597915

0.0408463	0.473	0.54	LOC400680	Hs.710554
0.0408523	0.473	1.39	SYT17	Hs.258326
0.0408736	0.473	2.31	PPT1	Hs.3873
0.0408852	0.473	1.53	ZDHHC13	Hs.188569
0.0410527	0.473	0.78	LOC286071	Hs.734739
0.0412733	0.473	1.54	RNASET2	Hs.529989
0.0412872	0.473	2.38	CNTNAP2	Hs.655684
0.0413079	0.473	0.69	IDS	Hs.460960
0.0413601	0.473	1.39	ABCF2	Hs.654958
0.0414367	0.473	0.58	LOC100505964	Hs.190267
0.0414377	0.473	0.69	PDZRN3	Hs.743998
0.0414818	0.473	0.66	C11orf1	Hs.17546
0.0415326	0.473	1.44	TMEM87B	Hs.656298
0.0415395	0.473	1.59	ARHGEF5	Hs.334
0.041577	0.473	1.59	CSF2RB	Hs.592192
0.0415996	0.473	0.76	TBX3	Hs.744016
0.0416056	0.473	0.83	RNGTT	Hs.567378
0.0416075	0.473	0.7	LOC100507506	Hs.665936
0.041662	0.473	1.34	ANKH	Hs.156727
0.0416954	0.473	0.71	MUC17	Hs.271819
0.0417213	0.473	0.8	PDSS1	Hs.558468
0.0418185	0.473	1.19	ARHGEF12	Hs.24598
0.0418316	0.473	2.24	SMAD7	Hs.465087
0.0418328	0.473	1.34	SLC16A3	Hs.500761
0.0419612	0.473	1.3	STK38	Hs.409578
0.0419718	0.473	1.52	DNAJC24	Hs.187269
0.0420035	0.473	1.76	GPHN	Hs.208765
0.0420391	0.473	1.34	PIGB	Hs.612814
0.0421079	0.473	1.49	MYO1C	Hs.286226
0.042121	0.473	1.46	SRGN	Hs.1908
0.0421958	0.473	0.38	ZNF709	Hs.631623
0.0421965	0.473	1.27	KLHL5	Hs.272251
0.0422192	0.473	1.35	GDAP1	Hs.168950
0.0422935	0.473	1.54	JAG1	Hs.224012
0.0422942	0.473	1.56	GRB2	Hs.444356
0.0423009	0.473	1.85	TNFAIP2	Hs.525607

0.0423302	0.473	1.35	ZNF561	Hs.720081
0.0423496	0.473	1.62	SLC38A6	Hs.200738
0.0424071	0.473	0.79	CARS2	Hs.508725
0.0424166	0.473	1.87	ARAP2	Hs.479451
0.0424215	0.473	1.81	MFAP4	Hs.296049
0.0424493	0.473	0.72	RAVER1	Hs.707428
0.0424552	0.473	1.46	DYRK4	Hs.439530
0.0424884	0.473	1.63	LAMB1	Hs.650585
0.042548	0.473	0.53	LOC503519	Hs.371380
0.0425631	0.473	1.93	MEF2C	Hs.649965
0.0425733	0.473	2.08	MFAP3L	Hs.593942
0.042574	0.473	1.61	TMEM177	Hs.439991
0.0425775	0.473	1.61	PRDX5	Hs.502823
0.0426076	0.473	1.47	TSPAN3	Hs.5062
0.0426323	0.473	1.41	NUB1	Hs.647082
0.0426436	0.473	1.95	SNHG8	Hs.535762
0.0427577	0.473	1.17	SMAD3	Hs.727986
0.0428018	0.473	1.37	RHBDD2	Hs.488827
0.0428038	0.473	0.72	CHRND	Hs.156289
0.0428273	0.473	1.44	UBL3	Hs.145575
0.0428274	0.473	0.62	LOC348817	Hs.669654
0.0428629	0.473	0.49	LOC441009	Hs.535004
0.0428659	0.473	0.67	C15orf57	Hs.654661
0.0429282	0.473	1.74	LAT	Hs.632179
0.0429579	0.473	0.38	MSX2	Hs.89404
0.0430325	0.473	1.4	LXN	Hs.478067
0.0430886	0.473	0.79	UBE3C	Hs.118351
0.0431014	0.473	0.68	MYO1F	Hs.465818
0.0431281	0.473	1.48	ZER1	Hs.147950
0.0431642	0.473	0.82	MEF2D	Hs.314327
0.043178	0.473	0.76	PXK	Hs.190544
0.0431992	0.473	0.72	STXBP4	Hs.35199
0.0432005	0.473	1.34	ELK3	Hs.46523
0.0432278	0.473	1.33	MAP4	Hs.517949
0.0432721	0.473	1.42	P4HA2	Hs.519568
0.0432977	0.473	0.68	NOS1AP	Hs.731942

0.0433382	0.473	1.59	GYG1	Hs.477892
0.0433419	0.473	2.52	HMGCS1	Hs.397729
0.0433893	0.473	1.66	YIPF6	Hs.82719
0.0434052	0.473	1.52	TCTN1	Hs.211511
0.0434458	0.473	0.69	ZNF687	Hs.186756
0.0434806	0.473	0.7	RARS2	Hs.485910
0.0434994	0.473	1.17	ENOX2	Hs.171458
0.0435314	0.473	1.66	STEAP3	Hs.647822
0.0436892	0.473	1.72	CELF2	Hs.309288
0.0437908	0.473	1.78	GCLM	Hs.315562
0.0438516	0.473	1.28	MYO5B	Hs.720076
0.043859	0.473	0.75	ZNF880	Hs.502314
0.0438779	0.473	1.41	HELZ	Hs.370140
0.0439144	0.473	1.51	GALNT11	Hs.647109
0.0439346	0.473	1.41	GMPR	Hs.484741
0.0439638	0.473	2.29	SERPINE1	Hs.414795
0.043984	0.473	0.66	MUC4	Hs.369646
0.0440172	0.473	2.05	SQLE	Hs.71465
0.044026	0.473	1.41	FBXO34	Hs.525348
0.0440316	0.473	1.36	NUDT3	Hs.188882
0.0440347	0.473	1.91	MS4A6A	Hs.523702
0.0440589	0.473	1.22	RB1CC1	Hs.196102
0.0440692	0.473	0.48	FGF9	Hs.111
0.0441002	0.473	0.59	BVES	Hs.221660
0.0441021	0.473	1.64	PTPN12	Hs.61812
0.0441175	0.473	0.73	C16orf52	Hs.498890
0.0441338	0.473	1.49	DDX18	Hs.363492
0.0442431	0.473	0.59	LIMD1	Hs.193370
0.0442586	0.473	1.51	HIST2H2AA3	Hs.530461
0.0442591	0.473	1.59	RAB31	Hs.99528
0.0442739	0.473	2.01	EPAS1	Hs.468410
0.0443033	0.473	1.92	IFITM3	Hs.374650
0.0443145	0.473	0.6	FAM135B	Hs.126024
0.0444335	0.473	1.36	FARP1	Hs.403917
0.0444526	0.473	3.37	PLA2G7	Hs.584823
0.0445066	0.473	0.81	AGAP4	Hs.656384

0.0445243	0.473	1.55	ZNF25	Hs.499429
0.0445426	0.473	0.71	HIST1H2BC	Hs.658713
0.0445437	0.473	1.32	CCP110	Hs.279912
0.0446348	0.473	0.78	DAGLB	Hs.487498
0.0446794	0.473	0.65	FKSG2	
0.0446961	0.473	1.41	CYCS	Hs.437060
0.0447038	0.473	1.52	MAFG	Hs.252229
0.0447099	0.473	1.48	RRBP1	Hs.472213
0.0447187	0.473	1.6	SLC39A11	Hs.221127
0.0447261	0.473	0.62	NUDT17	Hs.585066
0.0447336	0.473	1.29	CCND3	Hs.534307
0.044757	0.473	1.6	GAS7	Hs.462214
0.0447644	0.473	1.29	STAT5A	Hs.437058
0.0448198	0.473	1.63	RFFL	Hs.13680
0.0448477	0.473	1.33	GRB10	Hs.164060
0.0449185	0.473	1.3	MNT	Hs.626579
0.0449313	0.473	0.64	NDUFA4L2	Hs.744062
0.0449621	0.473	0.69	SLC12A4	Hs.10094
0.0450473	0.473	1.4	RCC2	Hs.380857
0.0451233	0.473	1.62	ARHGAP25	Hs.531807
0.0451555	0.473	1.38	DPAGT1	Hs.524081
0.0451597	0.473	1.35	CCR2	Hs.511794
0.0452442	0.473	0.81	ST3GAL6	Hs.148716
0.0452809	0.473	1.41	LMO2	Hs.34560
0.0453389	0.473	1.43	PARP2	Hs.409412
0.0453517	0.473	1.43	HAUS5	Hs.7426
0.045368	0.473	0.79	LOC645513	Hs.677546
0.0453836	0.473	0.75	LOC100507654	Hs.651545
0.0455034	0.473	1.49	BSDC1	Hs.353454
0.0455748	0.473	1.47	CSGALNACT2	Hs.657569
0.045598	0.473	1.4	USP30	Hs.486434
0.0456268	0.473	0.64	ZNF442	Hs.253193
0.0456388	0.473	0.75	TRAPPC10	Hs.126221
0.0456702	0.473	1.71	FCN3	Hs.333383
0.0456728	0.473	1.53	CD59	Hs.278573
0.0456799	0.473	1.46	BRI3BP	Hs.596464

0.0456997	0.473	0.68	TMEM241	Hs.137562
0.0457078	0.473	0.73	LOC100129722	Hs.656347
0.045746	0.473	1.91	SPRED1	Hs.525781
0.0457606	0.473	2.84	FKBP15	Hs.522351
0.0458128	0.473	1.3	PRPF31	Hs.515598
0.0458386	0.473	2.09	TIMP1	Hs.522632
0.0459117	0.473	0.68	ANGPT1	Hs.369675
0.0459387	0.473	1.54	IGSF6	Hs.530902
0.0459536	0.473	1.48	OSTM1	Hs.226780
0.0460112	0.473	2.86	SLC51A	Hs.630585
0.0460185	0.473	1.34	CLASP1	Hs.469840
0.046061	0.473	0.75	CCDC91	Hs.653125
0.0460656	0.473	2.14	HLA-B	Hs.654404
0.0460756	0.473	3.12	LAMP1	Hs.494419
0.0461055	0.473	1.63	LMTK2	Hs.444179
0.0461233	0.473	1.16	B3GALT6	Hs.284284
0.046131	0.473	1.57	BSG	Hs.501293
0.0461427	0.473	0.65	SULT1A1	Hs.567342
0.0461547	0.473	1.87	COX6A1	Hs.369624
0.0461906	0.473	1.3	CTDSPL	Hs.475963
0.0462302	0.473	1.69	OXSR1	Hs.475970
0.0462363	0.473	1.48	AKR1B1	Hs.521212
0.0462501	0.473	2.76	LGALS3	Hs.531081
0.0464156	0.473	1.49	CCDC174	Hs.517820
0.046426	0.473	0.62	KGFLP2	Hs.536967
0.0464627	0.473	0.64	LOC100271722	Hs.528519
0.0465545	0.473	2.4	CYBRD1	Hs.221941
0.0466081	0.473	0.6	SLC6A13	Hs.504398
0.0466352	0.473	1.3	NOB1	Hs.271695
0.0466531	0.473	0.59	CCDC144A	Hs.419859
0.0467002	0.473	2.33	FCHO2	Hs.741336
0.0467516	0.473	0.68	HSD17B1	Hs.654385
0.0467622	0.473	1.61	RPS27	Hs.546291
0.0468201	0.473	0.59	FLJ31485	Hs.288262
0.0468631	0.473	1.67	TBPL1	Hs.486507
0.0468945	0.473	1.43	ACTB	Hs.520640

0.0468993	0.473	0.57	CYP27C1	Hs.407639
0.0469404	0.473	12.93	XIST	Hs.529901
0.0469497	0.473	0.83	RALGAPA1	Hs.113150
0.0470314	0.473	1.6	TIGD7	Hs.161287
0.0470852	0.473	0.74	TRIM38	Hs.584851
0.0471029	0.473	2	EHD4	Hs.143703
0.0471158	0.473	0.85	RAB3IP	Hs.258209
0.0471813	0.473	0.65	MVB12B	Hs.162659
0.0472004	0.473	2.09	NEURL1B	Hs.91521
0.0472389	0.473	0.83	FAM105B	Hs.406335
0.0472785	0.473	0.83	LOC286272	Hs.648390
0.0472975	0.473	1.77	CD47	Hs.446414
0.0473026	0.473	1.63	OLFML3	Hs.9315
0.0473252	0.473	1.35	BOD1L1	Hs.444517
0.0473355	0.473	1.25	DYNLRB1	Hs.593920
0.047354	0.473	1.72	TSN	Hs.75066
0.0473741	0.473	1.78	CD52	Hs.276770
0.0474477	0.473	1.67	PFDN5	Hs.655327
0.0474611	0.473	0.79	PACS2	Hs.525626
0.0474658	0.473	1.56	COMMD6	Hs.508266
0.0474994	0.473	0.57	PSG7	Hs.709203
0.0475284	0.473	0.47	CBLN1	Hs.458423
0.0475774	0.473	0.75	SH3BGRL2	Hs.302772
0.0476154	0.473	1.21	ASXL1	Hs.374043
0.0476448	0.473	1.7	SEPN1	Hs.323396
0.0476459	0.473	1.29	HECA	Hs.197644
0.0476534	0.473	0.73	SP5	Hs.368802
0.047724	0.473	0.59	PPP1R9A	Hs.21816
0.0477291	0.473	1.5	PCM1	Hs.491148
0.0477962	0.473	0.84	RP9	Hs.326805
0.0478537	0.473	0.68	ANKRD28	Hs.335239
0.0478881	0.473	1.83	DPT	Hs.80552
0.0479599	0.473	2.19	LIPA	Hs.643030
0.0479858	0.473	0.78	RABL5	Hs.389104
0.0480057	0.473	1.23	STAT5B	Hs.743995
0.0480265	0.473	0.43	LINC00032	Hs.201554

0.0480297	0.473	1.72	PPP5C	Hs.654604
0.0480508	0.473	0.57	FAM105A	Hs.155085
0.0480575	0.473	2.62	GSTM1	Hs.301961
0.0480698	0.473	1.52	SUZ12	Hs.744174
0.0480829	0.473	1.42	ARSD	Hs.528631
0.0481004	0.473	0.64	BCL2L12	Hs.289052
0.0481182	0.473	1.67	TYROBP	Hs.515369
0.0481547	0.473	1.55	KLHL13	Hs.348262
0.048208	0.473	0.63	ATP6	
0.0482454	0.473	1.62	BTBD9	Hs.603858
0.0482532	0.473	2.47	RRM2	Hs.226390
0.0483134	0.473	0.55	LINGO2	Hs.650389
0.0483183	0.473	1.3	GNAQ	Hs.269782
0.0484046	0.473	0.7	DDX11	Hs.443960
0.0484191	0.473	1.35	UFM1	Hs.643655
0.0484918	0.473	1.4	STYXL1	Hs.11615
0.0484931	0.473	1.48	KANSL1L	Hs.282260
0.0485138	0.473	1.85	ACADVL	Hs.437178
0.0485724	0.473	1.58	FAM213A	Hs.500333
0.0485765	0.473	2.79	ARHGDIB	Hs.504877
0.0487014	0.473	1.52	PRAP1	Hs.15951
0.0487569	0.473	1.53	MAGOH	Hs.421576
0.0487937	0.473	0.45	CDS1	Hs.654899
0.0488085	0.473	0.62	ZFY-AS1	
0.0488208	0.473	1.43	UBTD2	Hs.131570
0.0488387	0.473	0.87	MED7	Hs.279902
0.0488877	0.473	1.75	TPM1	Hs.133892
0.0490408	0.473	2.74	REEP6	Hs.744099
0.0490422	0.473	2.11	GYG2	Hs.567381
0.0490668	0.473	1.73	ZNRF2	Hs.263912
0.0490724	0.473	1.24	PARN	Hs.253197
0.0491346	0.473	0.77	ELL	Hs.515260
0.0491762	0.473	0.68	FKSG49	
0.0491877	0.473	1.6	CHURC1	Hs.731537
0.0492124	0.473	1.85	MIPEP	Hs.507498
0.0492673	0.473	2.16	CSTB	Hs.695

0.0492885	0.473	1.35	GIMAP4	Hs.647101
0.0494204	0.473	1.43	TMEM62	Hs.511175
0.0494793	0.473	0.66	LOC100508120	Hs.188825
0.0495688	0.473	0.72	ZNF696	Hs.512740
0.0496199	0.473	2.53	GNG2	Hs.187772
0.0496714	0.473	1.76	CX3CR1	Hs.78913
0.0496771	0.473	1.56	GNG5	Hs.645427
0.0496782	0.473	1.54	ELF3	Hs.603657
0.0497248	0.473	0.78	POLR3C	Hs.591457
0.0497543	0.473	1.51	COPZ2	Hs.408434
0.0497588	0.473	0.81	FIP1L1	Hs.624245
0.0498304	0.473	0.66	LCMT2	Hs.200596
0.0498404	0.473	1.61	PGD	Hs.464071
0.0499172	0.473	1.29	SNRNP48	Hs.13366
0.0499215	0.473	1.2	FAM73A	Hs.437755
0.0499614	0.473	0.62	GAREML	Hs.187912
0.0499755	0.473	1.72	ANXA9	Hs.3346
0.0499776	0.473	1.74	ZNF148	Hs.592591
0.0499873	0.473	0.58	ANKFY1	Hs.696087

*ND, non-alcohol consumption group; LD, light alcohol consumption group
